# Decoding the mechanisms of chimeric antigen receptor (CAR) T cell-mediated killing of tumors: insights from granzyme and Fas inhibition

**DOI:** 10.1038/s41419-024-06461-8

**Published:** 2024-02-02

**Authors:** Melisa J. Montalvo, Irfan N. Bandey, Ali Rezvan, Kwan-Ling Wu, Arash Saeedi, Rohan Kulkarni, Yongshuai Li, Xingyue An, K M Samiur Rahman Sefat, Navin Varadarajan

**Affiliations:** William A. Brookshire Department of Chemical and Biomolecular Engineering, University of Houston, Houston, TX USA

**Keywords:** Apoptosis, Cell death and immune response

## Abstract

Chimeric antigen receptor (CAR) T cell show promise in cancer treatments, but their mechanism of action is not well understood. Decoding the mechanisms used by individual T cells can help improve the efficacy of T cells while also identifying mechanisms of T cell failure leading to tumor escape. Here, we used a suite of assays including dynamic single-cell imaging of cell-cell interactions, dynamic imaging of fluorescent reporters to directly track cytotoxin activity in tumor cells, and scRNA-seq on patient infusion products to investigate the cytotoxic mechanisms used by individual CAR T cells in killing tumor cells. We show that surprisingly, overexpression of the Granzyme B (GZMB) inhibitor, protease inhibitor-9 (PI9), does not alter the cytotoxicity mediated by CD19-specific CAR T cells against either the leukemic cell line, NALM6; or the ovarian cancer cell line, SkOV3-CD19. We designed and validated reporters to directly assay T cell delivered GZMB activity in tumor cells and confirmed that while PI9 overexpression inhibits GZMB activity at the molecular level, this is not sufficient to impact the kinetics or magnitude of killing mediated by the CAR T cells. Altering cytotoxicity mediated by CAR T cells required combined inhibition of multiple pathways that are tumor cell specific: (a) B-cell lines like NALM6, Raji and Daudi were sensitive to combined GZMB and granzyme A (GZMA) inhibition; whereas (b) solid tumor targets like SkOV3-CD19 and A375-CD19 (melanoma) were sensitive to combined GZMB and Fas ligand inhibition. We realized the translational relevance of these findings by examining the scRNA-seq profiles of Tisa-cel and Axi-cel infusion products and show a significant correlation between *GZMB* and *GZMA* expression at the single-cell level in a T cell subset-dependent manner. Our findings highlight the importance of the redundancy in killing mechanisms of CAR T cells and how this redundancy is important for efficacious T cells.

## Introduction

Chimeric antigen receptor (CAR) T cell therapy has made impressive advancements in the field of cancer immunotherapy within the last decade [1–3]. CAR T cell therapy has proven effective in the treatment of blood cancers such as lymphomas and leukemias with sustained remission, however, limitations exist such as treatment resistance and extending this technology to solid tumors [1–4]. To overcome these limitations, and engineer the next generation of immune cell therapies, a fundamental understanding of T cell cytotoxicity is essential. Two-photon microscopy studies in small animal models suggest that CAR T cells predominantly participate in direct cytotoxicity via cycles of direct engagement of tumor cells, killing, and detachment leading to apoptosis of tumor cells [5]. The mechanism of this direct cytotoxicity is however less established since T cells are known to use different approaches to induce tumor cell death such as cytokine release, tumor necrosis factor (TNF) trimerization, and exocytosis of cytotoxic enzymes. A fundamental understanding of the relative importance of these effector molecules and how they are impacted by the tumor cells is lacking [6–8].

Cytokines released from tumor-immune cell interactions, such as IFN-γ, do not induce direct killing, rather they have the capacity to mediate different cytotoxic pathways. IFN-γ signaling in tumor cells can promote antigen and MHC class I molecule presentation to increase sensitivity by T cell-mediated direct killing, and indirectly contribute to cytotoxicity through the upregulation of the cytokine TNF-α, a member of the TNF superfamily [9, 10]. Other members of the TNF superfamily can mediate direct cell death such as TNF-related apoptosis inducing-ligand (TRAIL) and Fas ligand (FasL) by recruiting the death-inducing signaling complex in tumor cells [7, 11]. FasL is enriched in T cells, as TRAIL is enriched in monocytes [12], making the relevancy of the Fas-FasL pathway an important CAR T cell killing mechanism.

Perhaps the most well-studied mechanism that T cells utilize to enable direct killing relies on serine proteases called granzymes [13–15]. Upon conjugation with a tumor cell, T cells release granzymes and perforin to enter tumor cells in a calcium-dependent manner [13–15]. Perforin coupled with calcium can form transient pores in the tumor cell membrane to allow the influx of granzymes into the cytosol and cleave apoptotic-inducing proteins [13–15]. In humans, five granzymes have been identified with granzyme B (GZMB) as the most widely studied [16–18]. It is hypothesized that GZMB is necessary for the induction of apoptosis in tumor cells and is synonymous with T cell cytotoxicity [14, 17, 18]. With GZMB as a key proponent in tumor cell apoptosis, focus has shifted to the role of the GZMB inhibitor, protease inhibitor-9 (PI9), in tumor resistance [19].

Here, we investigate the impact of PI9 overexpression in short-term CAR T killing of the liquid tumor line NALM6 and the solid tumor line SkOV3. We examine the quantity and quality of killing from the population to the single-cell level to show that CAR T- mediated killing is not impaired by PI9 overexpression, and that CAR T cells employ redundant cytotoxic mechanisms to compensate for reduced GZMB activity. To study these other mechanisms, in addition to PI9 overexpression, we block FasL and synthetically inhibit granzyme A (GZMA) in CAR T cells and we show that CAR T killing of NALM6 is severely dampened when both GZMA and GZMB are inhibited. In comparison, SkOV3 killing is significantly decreased when both GZMB and FasL are blocked, implying that CAR T cells employ redundancy in cytotoxic mechanisms to promote killing efficiency of tumor cells. We generalized our findings with lymphoma cell lines Raji and Daudi, and melanoma cell line A375-CD19 to show that our results are applicable across solid/liquid tumor types and further emphasize the redundancy of these cytotoxic mechanisms by mapping the co-expression of *GZMB* and *GZMA* in patient infusion products. These findings highlight the importance of multiple cytotoxic pathways that operate in facilitating T cell killing, and how these T cells can be engineered for optimal anti-tumor efficacy.

## Results

### Overexpression of PI9 does not impact long-term CAR T cell cytotoxicity

**Fig. 1:**
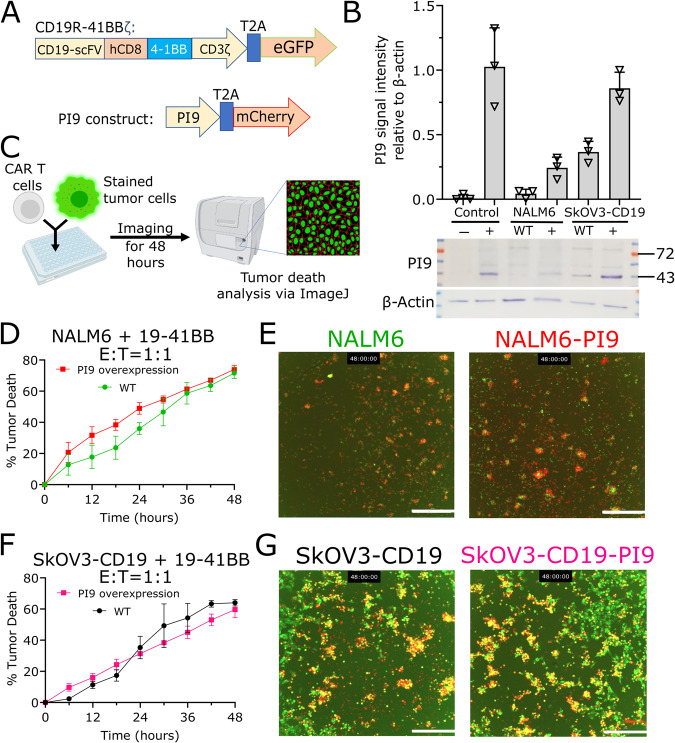
PI9 overexpression does not impact 19–41BB CAR T killing in cytotoxicity assays. **A** Design of CAR and PI9 constructs. The CAR construct contains an anti-CD19 single chain variable fragment (scFv), human CD8 transmembrane domain, 4–1BB costimulatory domain, CD3ζ activation domain, and a T2A peptide linker to enhanced GFP for cell sorting. **B** Western blot of PI9 protein expression in NALM6 and SkOV3-CD19 (wild-type and PI9-transduced). Negative control was MCF-7 and positive control was K562. The molecular weight of PI9 is 42 kDa. The bar graph represents the mean gray value of the SERPINB9 bands (*N* = 3) in relation to the loading control β-actin (*N* = 3). **C** Schematic of cytotoxicity assay. **D**, **E** Time-dependent cytotoxicity plot of 19–41BB CAR T cells against NALM6 and NALM6-PI9. Cytotoxicity percentage was corrected by subtracting the spontaneous target death percentage via tumor only control wells. Microscopy images at 48 h were extracted from one of the replicate wells. The tumor cells are green, the death marker is red and dead tumors appear yellow. The scale bar represents 300 μm. **F**, **G** Time-dependent cytotoxicity plot of 19–41BB CAR T cells against SkOV3-CD19 and SkOV3-CD19-PI9. Cytotoxicity percentage was corrected by subtracting the spontaneous target death percentage via tumor only control wells. Microscopy images at 48 h were extracted from one of the replicate wells. The tumor cells are green, the death marker is red and dead tumors appear yellow. The scale bar represents 300 μm [45–52,58, 59, 60]. * Cytotoxicity plots show the mean percentage of three replicate wells (*N* = 3) and the error bars show the SEM. Statistical testing for the cytotoxicity assay was performed at each time point using multiple Mann-Whitney tests.

We investigated whether overexpression of PI9 in NALM6 and SkOV3 tumor cell lines can attenuate cytotoxicity mediated by CD19R.4-1BBζ CAR T cells (shortened to 19-41BB) (Fig. 1A). To ensure uniform antigen expression, we previously transduced the SkOV3 cell line to express the CD19 surface receptor and verified CD19 expression (>95%) by flow cytometry (Fig. S1) [20]. We transduced NALM6 and SkOV3-CD19 with PI9, which yielded the two cell lines: NALM6-PI9 and SkOV3-CD19-PI9. We verified the overexpression of PI9 in both these cell lines via Western blotting and indirect flow cytometry (Figs. 1B, S2). Since one of the primary mechanisms of GZMB activation is to directly activate pro-caspase 3 in tumor cells, we confirmed via western blotting that the expression of pro-caspase 3 was not different between NALM6 and NALM6-PI9 and SkOV3-CD19 and SkOV3-CD19-PI9 (Fig. S3).

We manufactured 19-41BB CAR T cells from two healthy donors, and phenotyping confirmed high CAR expression (64-74%) and balanced CD4/CD8 distributions (Fig. S4). To assess the impact of PI9 overexpression in long-term cytotoxicity mediated by T cells, we co-cultured the CAR T cells as effectors and tumor cells as targets to visualize CAR T cytotoxicity over 48 h (E:T = 1:1, Fig. 1C). Surprisingly, the rate of killing mediated by 19-41BB CAR T cells against either NALM6 or NALM6-PI9 did not differ over time (Fig. 1D, E). Similarly, the rate of killing by 19-41BB CAR T cells was no different between SkOV3-CD19 and SkOV3-CD19-PI9 cells (Fig. 1F, G). These observations were also consistent at much lower effector-target ratios of 1:5 and 1:10 (Fig. S5). To independently confirm the lack of impact by GZMB inhibition on cytotoxicity against tumors cells, we pre-treated CAR T cells with a small molecule inhibitor of GZMB, Z-AAD-CMK, and repeated the cytotoxicity assay with the wild-type NALM6 and SkOV3-CD19 incubated with excess Z-AAD-CMK (Fig. S6A). Consistent with our PI9 overexpression data, we observed no significant differences in killing over time (Fig. S6B, C). Collectively, these findings illustrate that inhibiting GZMB in CAR T cells does not impact long-term killing capacity.

### PI9 overexpression in NALM6 does not affect quantity or quality of CAR T cell- mediated killing at the single-cell level in short-term assays

**Fig. 2:**
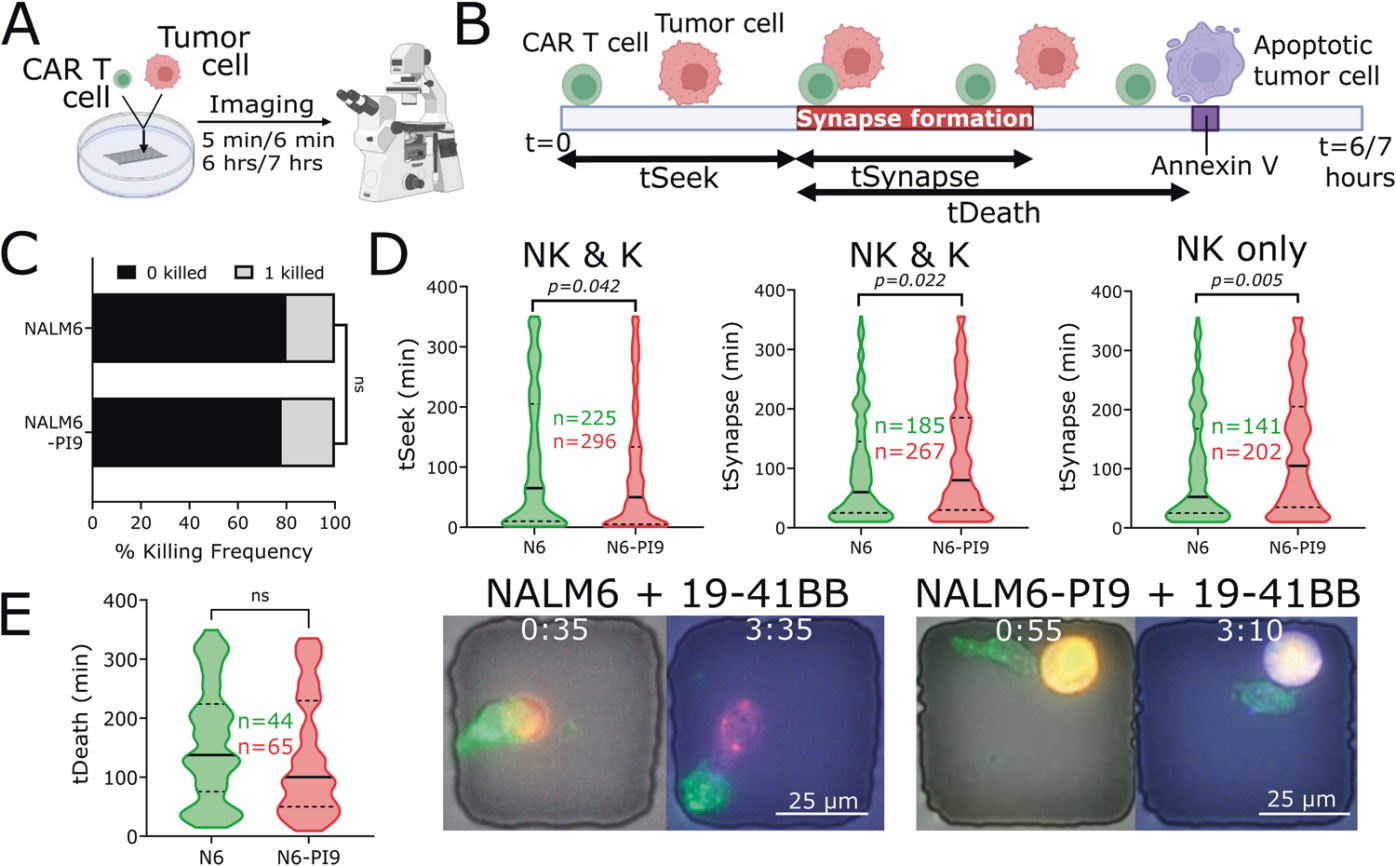
PI9 overexpression does not impact single-cell kinetics or killing frequencies by 19–41BB CAR T cells. **A** Schematic of TIMING assay. **B** Schematic of kinetic measurements from TIMING assay. **C** Bar graph showing killing frequencies of NALM6 (N = 44/225) and NALM6-PI9 (*N* = 65/296). **D** Violin plots showing seek times and synapse duration of non-killer (NK) and killer (K) CAR T cells against NALM6 and NALM6-PI9. Bold line represents the median and the dotted lines represent the upper/lower quartiles. **E** Violin plot showing the time of death for NALM6 and NALM6-PI9. Micrographs shown for NALM6 and NALM6-PI9 killing by 19-41BB CAR T cells. First frame shows initial conjugation and second frame shows target death. Target is labeled red, the effector green, and the death marker blue. Time above the micrographs is denoted as (H:MM). * For bar graphs, statistical testing was performed using the chi-square test of independence. For violin plots, statistical testing was performed using the unpaired t-test.

To rule out the role of T cell cooperation from the cytotoxicity assays and map short-term killing differences, we next explored the impact of PI9 overexpression at the single-cell level. We use time-lapse imaging microscopy in nanowell grids (TIMING) over 6–8 h of individual T cells against the same panel of tumor cell lines (Fig. 2A, B). At an E:T of 1:1, we did not observe significant differences in 19–41BB CAR T cell killing frequencies of NALM6 and NALM6-PI9, and this observation was conserved with both CD8 and CD4 CAR T cells as effectors (Figs. 2C and S7). The lack of change in single-cell killing frequencies mirror our observations from the population assays: PI9 overexpression does not impact CAR T cytotoxicity against NALM6 (Fig. 1E).

Next, we assessed the kinetics of the T cell-tumor cell interactions. We observed that individual CAR T cells spend significantly more time conjugated to the NALM6-PI9 cells (115 ± 6 min) than NALM6 cells (94 ± 6 min), but the longer conjugation times did not delay time needed to induce target death (Fig. 2D, E, Movie 1, 2). To understand why the longer conjugation times do not delay the time to killing, we separated the conjugation times based on events where the target died (killer) and events where the target did not die (non-killer) and show that the increased conjugation times of CAR T cells with NALM6-PI9 cells is largely because of non-killer T cells remaining conjugated to the NALM6-PI9 tumors without inducing death (Fig. 2D). In aggregate, these results suggest that GZMB inhibition by PI9 does not alter the frequency or kinetics of CAR T cell-mediated killing of tumor cells.

### Single-cell reporter of granzyme activity confirms reduced GZMB activity upon PI9 overexpression in tumor cells

**Fig. 3:**
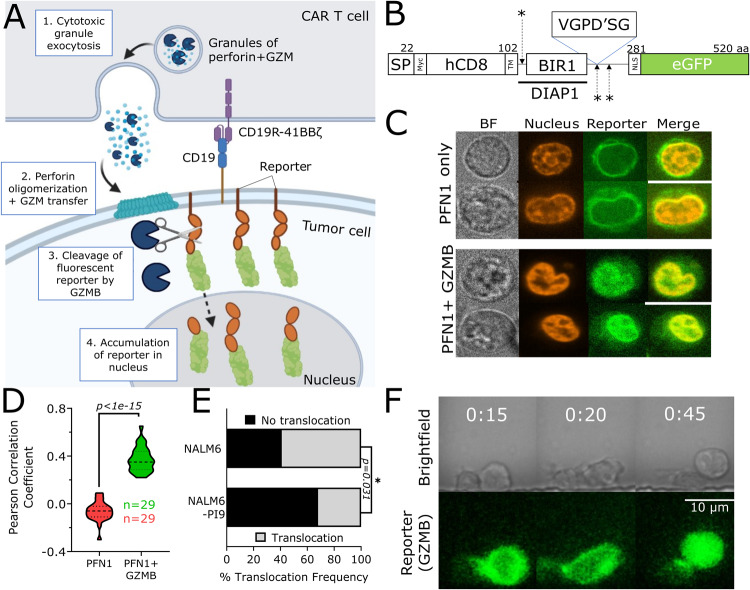
Reporter assay shows reduced granzyme B activity associated with PI9 overexpression in NALM6. **A** Overview of mechanism of fluorescent reporter model. **B** Design of granzyme B reporter vector construct containing a granulocyte- macrophage colony-stimulating factor leader sequence, Myc tag for detection, human CD8 transmembrane domain, Drosophila inhibitor of apoptosis protein 1 (DIAP1) domain, three protease sites, and a nuclear localization signal ending with an enhanced GFP. The DIAP1 section contains a BIR1 domain that enhances caspase recognition and the asterisks represent the protease sites. The protease sites from P4’-P2 contain the amino acid sequence VGPD’SG. **C** Confocal images of reporter translocation in single cells using purified perforin and granzyme B. Brightfield shows the phase image, nucleus panel shows DRAQ5 nuclear staining, reporter panel shows the reporter fluorescence, and the merged panel shows the nucleus and reporter fluorescence overlap. Scale bar represents 10 µm. **D** Violin plot showing the Pearson correlation coefficient between CY5 (nuclear stain) and AF488 (reporter) with and without purified granzyme B. The heavy dotted line represents the median and the lighter dotted lines represent the upper and lower quartiles. **E** Bar plot showing translocation frequencies of granzyme B reporter in NALM6- GBR (*N* = 19/32) and NALM6-PI9-GBR (*N* = 10/31) killing events. **F** Time-lapse images of granzyme B reporter translocation induced by 19–41BB CAR T cells. Time above the micrographs is denoted as (H:MM). * For violin plots, statistical testing was performed using the Mann-Whitney test. For bar graphs, statistical testing was performed using the chi-square test of independence.

Since our assays demonstrated that PI9 does not alter the dynamics or the frequency of killing mediated by CAR T cells, we next designed a fluorescent reporter to directly visualize GZMB activity at the single-cell level within tumor cells. We designed the reporter to be a substrate of GZMB; successful proteolysis of the membrane-tethered GZMB reporter will cause translocation of the fluorescent protein to the nucleus, facilitated by the nuclear localization signal (Fig. 3A, B). We transduced NALM6 and NALM6-PI9 with the GZMB fluorescent reporter to yield NALM6-GBR (GZMB reporter) and NALM6-PI9-GBR. To assess specificity of the protease sites, we incubated our reporter cell lines with purified perforin and mature form of GZMB. We confirmed that nuclear translocation of GFP required both GZMB and perforin; GFP was localized to the membrane in the absence of GZMB (Fig. 3C, D).

We next tested the fluorescent reporter cell lines upon co-incubation with CAR T cells. Using a TIMING chip, we co-loaded our reporter cell lines with 19–41BB CAR T cells and imaged them for 6 h. To quantify nuclear translocation, we trained and validated a nuclear segmentation module to assess reporter translocation without nuclear staining (Fig. S8). Next, we quantified the frequency of reporter translocation in both NALM6-GBR and NALM6-PI9-GBR upon killing by 19–41BB CAR T cells (Fig. 3E, F). We show a significantly lower frequency of reporter activity in NALM6-PI9-GBR killing events compared to NALM6-GBR confirming that PI9 overexpression directly inhibits GZMB activity in the tumor cell cytosol. By quantifying both PI9 inhibition and killing at single-cell resolution, we are able to demonstrate that while tumor cell PI9 overexpression does inhibits GZMB activity as expected, this inhibition surprisingly does not result in reduced cytotoxicity of CAR T cells.

### Blocking FasL does not impact single-cell CAR T-mediated killing of NALM6 cells

**Fig. 4:**
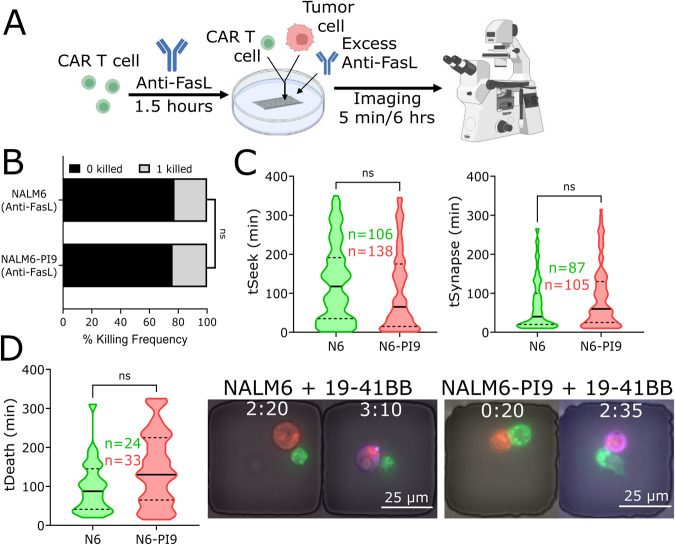
Overexpressing PI9 and blocking FasL does not impact killing of NALM6 by 19–41BB CAR T cells. **A** Schematic of blocking FasL on 19–41BB CAR T cells in TIMING assay. **B** Bar graph showing killing frequencies of NALM6 (*N* = 24/87) and NALM6-PI9 (*N* = 33/105) with FasL blocked. **C** Violin plots showing seeking times and synapse duration for NALM6 and NALM6-PI9. Bold line represents the median and the dotted lines represent the quartiles. **D** Violin plot showing the time of death for NALM6 and NALM6-PI9. Micrographs shown for NALM6 and NALM6-PI9 killing by 19–41BB CAR T cells. First frame shows initial conjugation and second frame shows target death. Target is labeled red, the effector green, and the death marker blue. Time above the micrographs is denoted as (H:MM). * For bar graphs, statistical testing was performed using the chi-square test of independence. For violin plots, statistical testing was performed using the unpaired t-test.

Since the Fas-FasL axis can be an important contributor to CAR T cell toxicity, we next investigated if FasL-mediated killing could explain why CAR T cell-mediated killing was unaltered upon GZMB inhibition [21]. We stained for surface Fas expression on both NALM6 and NALM6-PI9, and flow cytometry revealed relatively low expression on both cell lines (Fig. S9). To rule out if Fas expression is a major contributor to tumor cell death upon FasL ligation by CAR T cells, we pre-incubated 19–41BB CAR T cells with an anti-FasL antibody and loaded them on a TIMING chip with NALM6 or NALM6-PI9 cells and excess FasL antibody (Fig. 4A). We observed negligible differences in NALM6 and NALM6-PI9 killing frequencies and kinetics with anti-FasL blocking, suggesting that simultaneously blocking FasL and inhibiting GZMB did not affect short-term killing by CAR T cells (Fig. 4B–D). In conjunction with our previous experiments where we show that inhibition of GZMB alone does not impair killing of NALM6 tumor cells, here we show combined blockade of the Fas pathway and GZMB inhibition does not impair killing of NALM6 tumor cells, suggesting the importance of alternate cytotoxic mechanisms that can be responsible for CAR T cell-mediated cytotoxicity.

### Blocking GZMA impacts single-cell CAR T-mediated killing in NALM6

**Fig. 5:**
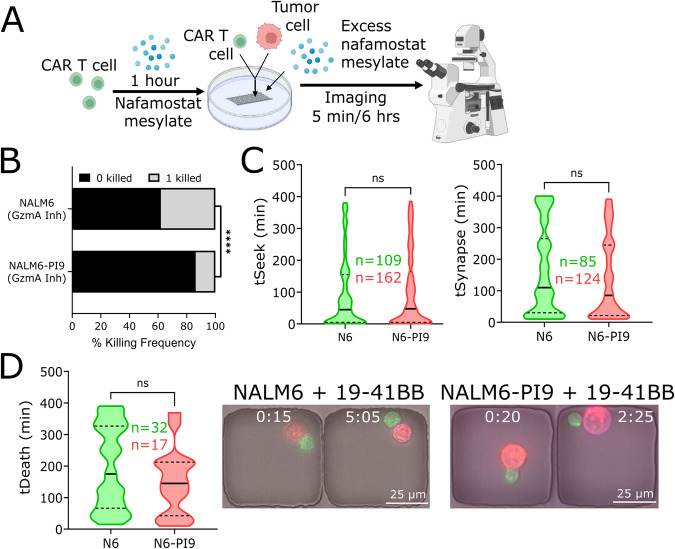
GZMA inhibition significantly decreases NALM6-PI9 killing by 19–41BB CAR T cells. **A** Schematic of inhibiting GZMA in TIMING assay. **B** Bar graph showing killing frequencies of NALM6 (*N* = 32/85) and NALM6-PI9 (*N* = 17/124) with GZMA inhibition. **C** Violin plots showing seeking times and synapse duration and death in NALM6 and NALM6-PI9. Bold line represents the median and the dotted lines represent the quartiles. **D** Violin plot showing the time of death for NALM6 and NALM6-PI9. Micrographs shown for NALM6 and NALM6-PI9 killing by 19–41BB CAR T cells. First frame shows initial conjugation and second frame shows target death. Target is labeled red, the effector green, and the death marker blue. Time above the micrographs is denoted as (H:MM). * For bar graphs, statistical testing was performed using the chi-square test of independence. For violin plots, statistical testing was performed using the unpaired t-test.

As we previously discovered that CAR T cell killing of NALM6 tumor cells remains unaffected by GZMB inhibition and FasL blocking, we next investigated the contribution of GZMA in CAR T-mediated killing. We prioritized exploring GZMA over GZMH, GZMK, and GZMM since RNA transcripts for *GZMA* and *GZMB* in patient infusion products were the highest and most similar of all granzymes (data not shown). We pre-incubated our 19-41BB CAR T cells with nafamostat mesylate (inhibitor of GZMA), and loaded them on our TIMING chip with NALM6 or NALM6-PI9 in the presence of excess nafamostat mesylate (Fig. 5A). We observed a significant decrease in the magnitude NALM6-PI9 killing compared to NALM6 with no differences in the kinetics of tumor cell conjugation or killing (Fig. 5B–D). Collectively, these results suggest a role for GZMA-like serine proteases in 19-41BB CAR T cell cytotoxicity against NALM6 tumor cells.

### SkOV3-CD19 is sensitive to Fas-mediated death by CAR T cells, but not granzymes

**Fig. 6:**
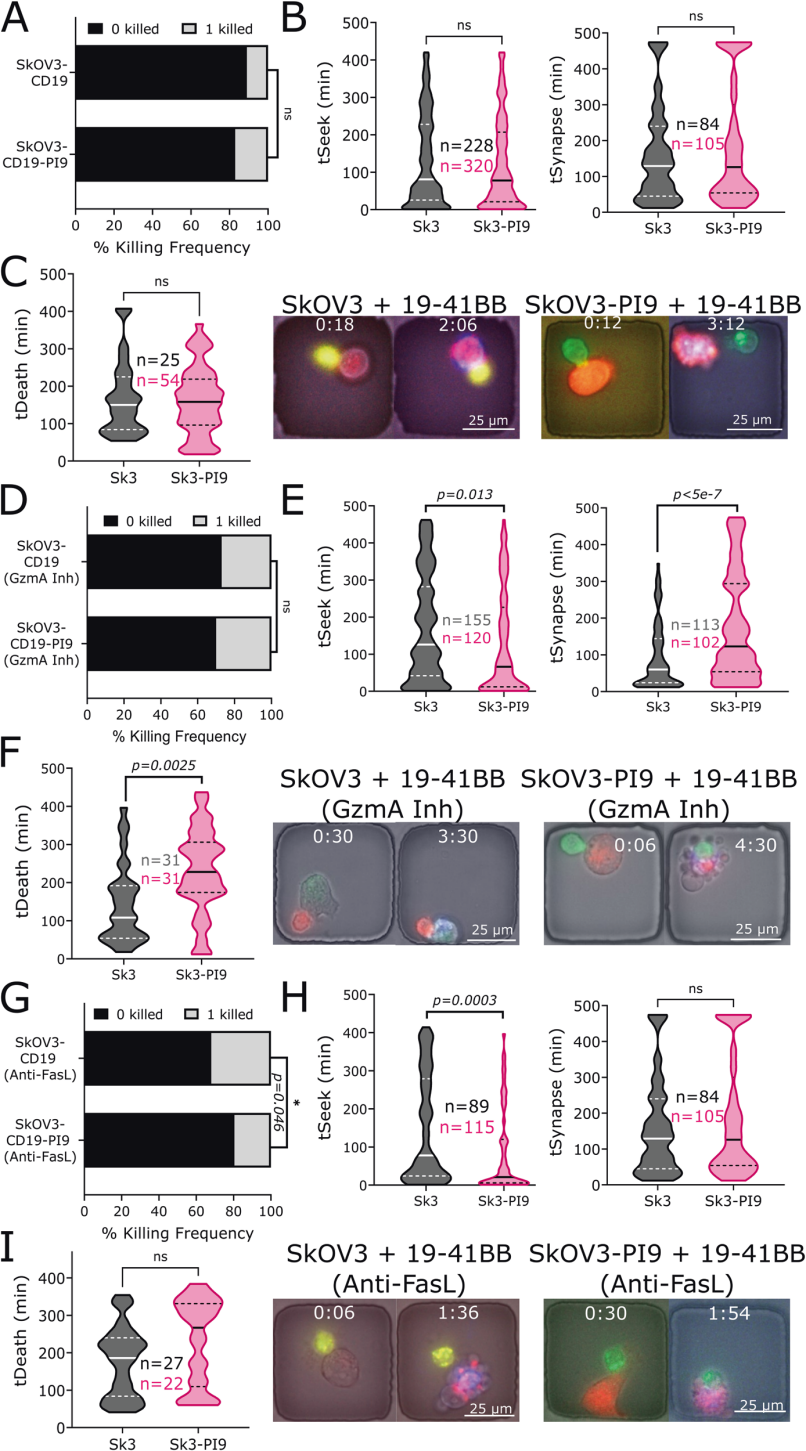
SkOV3-CD19 killing is sensitive to Fas-mediated killing by 19-41BB CAR CAR T cells. **A** Bar graph showing killing frequencies of SkOV3-CD19 (*N* = 25/208) and SkOV3- CD19-PI9 (*N* = 54/290). **B** Violin plots showing seeking times and synapse duration for SkOV3-CD19 and SkOV3-CD19-PI9. Bold line represents the median and the dotted lines represent the quartiles. **C** Violin plot showing the time of death for SkOV3-CD19 and SkOV3-CD19-PI9. Micrographs shown for SkOV3-CD19 and SkOV3-CD19-PI9. killing by 19–41BB CAR T cells. First frame shows initial conjugation and second frame shows target death. Target is labeled red, the effector green, and the death marker blue. Time above the micrographs is denoted as (H:MM). **D** Bar graph showing killing frequencies of SkOV3-CD19 (*N* = 31/113) and SkOV3- CD19-PI9 (*N* = 31/102) with nafamostat mesylate (NaMe) treatment. **E** Violin plots showing seeking times and synapse duration for SkOV3-CD19 and SkOV3-CD19-PI9 by 19–41BB CAR T cells treated with NaMe. Bold line represents the median and the dotted lines represent the quartiles. **F** Violin plot showing the time of death for SkOV3-CD19 and SkOV3-CD19-PI9. Micrographs shown for SkOV3-CD19 and SkOV3-CD19-PI9 killing by 19–41BB CAR T cells treated with NaMe. First frame shows initial conjugation and second frame shows target death. Target is labeled green, the effector red, and the death marker blue. Time above the micrographs is denoted as (H:MM). **G** Bar graph showing killing frequencies of SkOV3-CD19 (*N* = 27/84) and SkOV3- CD19-PI9 (*N* = 22/105) with anti-FasL treatment. **H** Violin plots showing seeking times and synapse duration for SkOV3-CD19 and SkOV3-CD19-PI9 by 19–41BB CAR T cells treated with Anti-FasL. Bold line represents the median and the dotted lines represent the quartiles. **I** Violin plot showing the time of death for SkOV3-CD19 and SkOV3-CD19-PI9. Micrographs shown for SkOV3-CD19 and SkOV3-CD19-PI9 killing by 19–41BB CAR T cells treated with Anti-FasL. First frame shows initial conjugation, and second frame shows target death. Target is labeled red, the effector green, and the death marker blue. Time above the micrographs is denoted as (H:MM). * For bar graphs, statistical testing was performed using the chi-square test of independence. For violin plots, statistical testing was performed using the unpaired t-test for *N* > 25.

Based on the importance of GZMA in NALM6 killing, we next wanted to explore whether the same mechanism contributes to killing of SkOV3-CD19 tumor cells. Our population killing assay showed no significant differences in killing with PI9 overexpression (Fig. 1E, F), so we examined SkOV3-CD19 killing at the single-cell level. From the TIMING assay, we observed no differences in either the magnitude or the dynamics of killing mediated by CAR T cells comparing SkOV3-CD19 and SkOV3-CD19-PI9 (Fig. 6A–C, Movie 3, 4). We confirmed that similar to killing of NALM6 tumor cells, both CD8 and CD4 CAR T cells were capable of killing these tumor targets at the single-cell level (Fig. S9). We next assessed the impact of combined inhibition of GZMA and GZMB in CAR T cells on SkOV3 killing. We observed no differences in quantity of killing but show a clear impact on the kinetics associated with tumor killing (Fig. 6D–F). Here we observe a significant increase in both the duration of the synapse and killing time associated with PI9 overexpression and GZMA inhibition. These results suggest that the combined inhibition of both granzymes does not impact the magnitude of the killing but significantly delays the kinetics of killing mediated by CAR T cells. Since it is well-known from both single-cell assays and population-level assays that death induced by Fas-FasL is associated with slower kinetics of killing [22], we next explored the impact of FasL inhibition.

To explore the contribution of the FasL-Fas pathway, we stained for Fas expression and both SkOV3-CD19 and SkOV3-CD19-PI9 tumor cells showed high expression by flow cytometry (Fig. S10). When FasL was blocked on CAR T cells, we observed a significant reduction in killing frequencies against SkOV3-CD19-PI9 compared to SkOV3-CD19, demonstrating that the FasL-Fas axis can be a significant contributor to CAR T cell- mediated cytotoxicity against solid tumor targets (Fig. 6G–I).

In addition to cytotoxins, secreted cytokines can directly increase the sensitivity of tumor cells to T cell-mediated cytotoxicity. Accordingly, we investigated if two important cytokines, IFN-γ and TNF-α can contribute positively to the killing mediated by CAR T cells. We confirmed that co-incubation of 19–41BB CAR T cells with NALM6, NALM6-PI9, SkOV3-CD19, or SkOV3-CD19-PI9 tumor cells led to the secretion of IFN-γ in the supernatants (Fig. S11). We stained the IFN-γ and TNF-α receptors on the tumor cells and flow cytometry revealed that the PI9 overexpressing cell lines express IFNGR1 and TNFAR1 receptors at lower frequencies than the parental tumor cells (Fig. S12). To test if interaction between the cytokine and its cognate cytokine receptor had any impact on the direct cytotoxicity mediated by the CAR T cells, we performed a cytotoxicity assay blocking IFN-γ and TNF-α (Fig. S13A). We show no differences in killing frequencies between the parental and PI9 overexpressing tumor cells, highlighting that GZMB inhibition by PI9 and concomitant blockade of both IFN-γ and TNF-α cytokines is not sufficient to impair CAR T cell cytotoxicity (Fig. S13B, C).

**Fig. 7:**
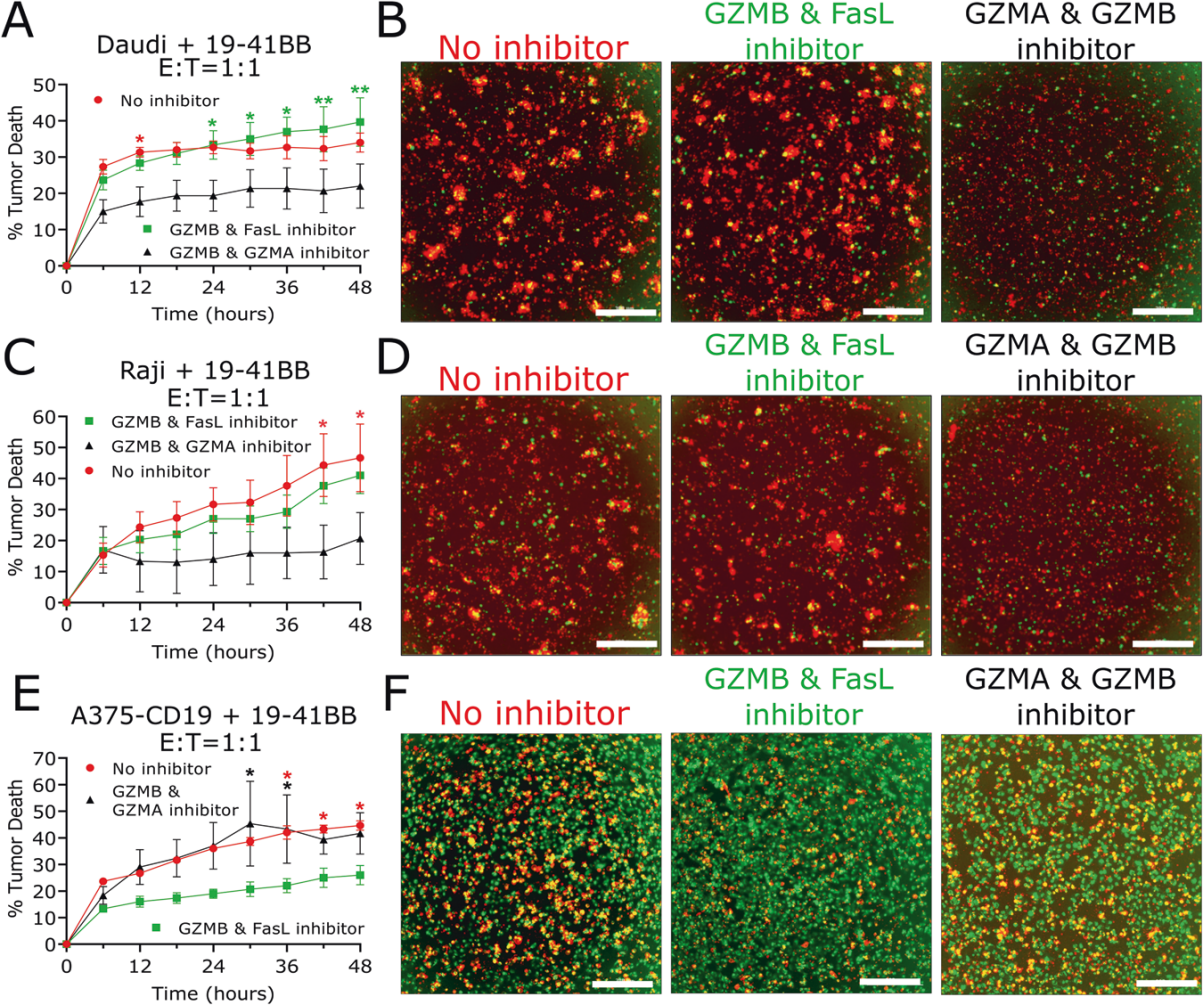
Combined inhibition of multiple pathways is required for altering the magnitude of killing mediated by CAR T cells. **A**, **C**, **E** Time-dependent cytotoxicity plot of 19–41BB CAR T cells treated with cytotoxic inhibitors against tumor cell lines. Cytotoxicity percentage was corrected by subtracting the spontaneous target death percentage via control wells. The red data points represent T cells without treatment, the green data points represent T cells incubated with GZMB and FasL inhibitors, and the black data points represent T cells incubated with GZMA and GZMB inhibitors. Asterisks represent statistical testing between the bottom curve and the timepoints corresponding to the color of the asterisk. **B**, **D**, **F** End point microscopy images from each condition. Tumor cells are green, the death marker is red, and dead tumors appear as yellow. The scale bar represents 300 μm. * Cytotoxicity plots show the mean percentage of three replicate wells (*N* = 3) and the error bars show the SEM. Statistical testing for the cytotoxicity assay was performed at each time point using Tukey’s method for multiple comparisons.

To generalize our findings to other cell lines, we performed cytotoxicity assays with CAR T cells against Daudi, Raji, and A375-CD19 cells in the presence of the cytotoxic inhibitors. We incubated our cell lines with excess inhibitor to ensure complete GZMB inhibition where endogenous PI9 levels are rendered negligible. Consistent with our results with NALM6, we show that both lymphoma cell lines (Daudi and Raji) are sensitive to combined GZMA and GZMB inhibition, most likely attributed to their minimal Fas surface expression (Fig. 7A–D) [12]. Also consistent with our results with SkOV3-CD19, we show that A375-CD19 melanoma cells are sensitive to combined FasL and GZMB inhibition, since it is already known that Fas expression of A375 is comparable to SkOV3 (Fig. 7E, F) [12]. These results are indicative of a model in which redundancy in granzymes facilitates killing against leukemias/lymphomas with low Fas expression whereas redundancy between GZMB and FasL facilitates killing of solid tumor targets with high Fas expression.

### Single-cell RNAseq highlights correlation between *GZMA* and *GZMB* in infusion products

**Fig. 8:**
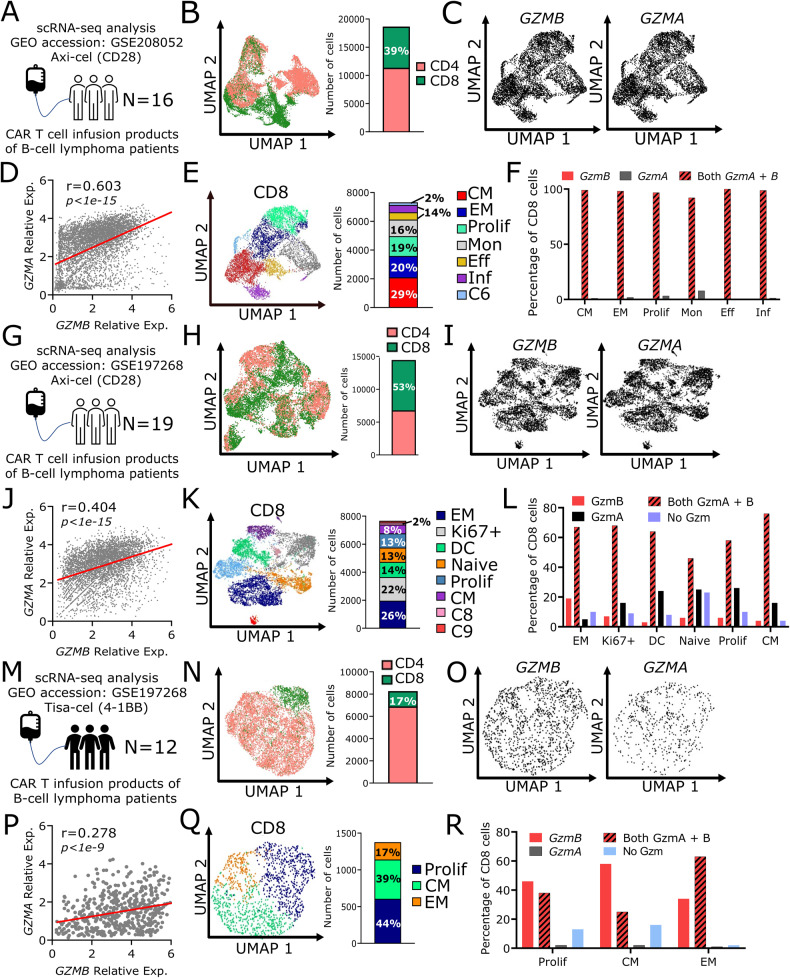
scRNA-seq of patient infusion products show that CD8^+^CAR T cells co-express *GZMB* and *GZMA* in multiple T cell subsets. **A**, **G**, **M** Overview of scRNAseq data cohort. **B**, **H**, **N** Uniform manifold approximation plot (UMAP) showing CD4/CD8 distribution across all patient CAR T cells. Percentages summarized as a bar plot (right). **C**, **I**, **O** UMAP showing distribution of *GZMB* and *GZMA* expression in CD8^+^CAR T cells. **D**, **J**, **P** Scatter plot showing relative expression levels of *GZMB* and *GZMA*. The Pearson correlation coefficient (r) was calculated with the p values below. **E**, **K**, **Q** UMAP showing distribution of CD8^+^CAR T cells from unsupervised clustering. Percentages summarized as a bar plot (right). Clusters with less than 100 cells were omitted from cluster classification (i.e C6; C8 and C9). **F**, **L**, **R** Bar plot showing percentage of CD8 CAR T cells by cluster expressing *GZMB* only (red), both *GZMB* and *GZMA* (striped), *GZMA* only (black), and neither *GZMB* or *GZMA* (blue).

To contextualize the relationships between the cytotoxic pathways explored in this study within patient infusion products, we profiled CD3^+^CAR^+^ single cells from three sets of patient infusion products (2 Axi-cel and 1 Tisa-cel) including one of our own datasets (Fig. 8A, G, M) [23, 24]. As the products are derived from patients with diffuse large B-cell lymphoma, we focused on the redundancy between *GZMB* and *GZMA*. We subset the infusion products based on CD4/CD8 expression and show that Axi-cel products (7–76%, 15–95%) have a higher frequency of CD8 T cells compared to Tisa-cel products (2–20%) (Fig. 8B, H, N). Focusing on the CD8^+^ cells, we calculated the correlation between detectable *GZMA* and *GZMB* transcripts within the same cells. Single cells derived from both Axi-cel infusion products showed a strong correlation between *GZMA* and *GZMB* expression (Fig. 8D, J) whereas the correlation between these same transcripts was weaker within the Tisa-cel derived single cells (Fig. 8P). To understand how the differentiation status of the CAR T cells influenced this correlation, we performed unsupervised clustering on the CD8 cells from each of these infusion products. We identified six major clusters for our Axi-cel product (Central Memory, Effector Memory (EM), Proliferative, Monocyte-like, Effector, Inflammatory), six major clusters for the Supplementary Axi-cel dataset (EM, Ki67^+^, Dendritic Cell-like, Naïve, Proliferative, Central Memory), whereas the Tisa-cel products yielded three clusters (Proliferative, Central Memory, EM) [Fig. 8E, K, Q, S14]. In both the Axi-cel and Tisa-cel CD8 CAR T cells, >60% of the EM cells expressed both *GZMB* and *GZMA*, consistent with the role of T_EM_ cells as memory population with immediate cytolytic capability catalyzing anti-tumor killing (Fig. 8F, L) [25]. Not surprisingly, >60% CD8 T cells from both Axi-cel products showed expression of both *GZMA* and *GZMB* at the single-cell level, consistent with the known immunobiology of the CD28-endodomain CAR in promoting effector functionality (Fig. 8C–I) [26–28]. By contrast, within the Tisa-cel CD8 CAR T cells, only 20% of the less differentiated T_CM_ cells showed co-expression of *GZMA* and *GZMB*, consistent with the immunobiology of the 41BB-endodomain CAR in promoting longevity but not immediate cytotoxicity (Fig. 8O, R) [26–29]. To test if Tisa-cel derived CAR T cells express *GZMA* after infusion into the patients, we profiled the RNAseq of Tisa-cel infusion products 10 days post-infusion. The expression of *GZMA* was significantly higher than *GZMB* in CD8^+^ CAR T cells further emphasizing the compensatory role of *GZMA* (Fig. S15). In summary, the scRNA-seq data from infusion products demonstrate that CD8 T_EM_ CAR T cells express multiple granzymes and this allows for redundancy in cytotoxic pathways, making them potent effectors even against PI9 expressing tumor cells.

## Discussion

Our study explores the main cytotoxic mechanisms associated with CAR T-mediated death through a series of studies utilizing both healthy donor derived CAR T cells and patient infusion products. We demonstrate that surprisingly, CAR T cell-mediated cytotoxicity is not significantly impacted by overexpression of PI9 within tumor cells; rather, redundancy in cytotoxic pathways ensures that compensatory cytotoxic mechanisms including GZMA for liquid tumor targets and FasL for solid tumor targets preserve CAR T cell cytotoxicity.

To extend our finding more broadly, we examine the relative efficacy of these CAR T cell killing mechanisms in diverse tumors. CAR T cell therapies have also been approved for multiple myeloma (MM) [30, 31]. It is well-known that interleukin-6 functions as a key growth factor in MM by rendering tumor cells insensitive to Fas-mediated apoptosis [32]. For this reason, similar to our results with lymphomas, we anticipate that GZMB and GZMA will be the principal axes of cytotoxicity of BCMA CAR T cells targeting MM. In solid tumors, immunohistochemical staining data from the Human protein atlas shows that Fas expression is detectable in several solid tumors (https://www.proteinatlas.org/ENSG00000026103-FAS/pathology). Thus, staining for Fas expression within solid tumors might be straightforward assay to ascertain sensitivity to CAR T cell-mediated cytotoxicity.

The role of PI9 within the immune system is crucial; cells endogenously express PI9 as a protection shield against cytotoxic lymphocytes during maturation, however, tumors have been observed to overexpress PI9 to prevent killing by immune cells [19, 33–41]. Immunohistochemical analysis of cancer patient tissues suggests that overexpression of PI9 is responsible for tumor progression in prostate, lung, and melanoma cancers [38, 40, 42]. These observations were substantiated by a series of both in vitro and in vivo studies that investigated the killing mediated by CTLs against a series of murine and human tumor cell lines wherein PI9 expression was associated with resistance to CTL killing and was consequently described as an immune escape mechanism [37, 41]. Other in vitro studies also imply that PI9 negatively controls cytotoxic lymphocyte killing of HeLa and leukemic cells [34, 43]. The reason PI9 overexpression is associated with better tumor survival in a variety of cancer types is likely attributed to its fast-acting irreversible inhibition of GZMB and its consequent deactivation [33, 37, 41, 44]. GZMB is characterized by its fast kinetics as it cleaves substrates 10–100 fold times higher than other highly expressed granzymes, however, the affinity between GZMB-PI9 is 100 times higher than these same granzymes, rendering GZMB inactive upon inhibition by PI9 (Supplementary Table 1) [45–52]. The key question we ask is whether this PI9 inhibition of GZMB is sufficient to impact CAR T cytotoxicity. As the data with our genetic reporter based assays confirm, overexpression of PI9 does directly reduce GZMB activity in tumor cells. The overall cytotoxicity mediated by CAR T cells is however unaffected primarily due to redundancy in mechanisms of cytotoxicity.

Our results with CAR T cells complement data from studies investigating the cytotoxicity of in vitro derived CTLs targeting virus-specific epitopes presented in HLA-A2 lymphoma cell lines expressing high levels of PI9 [53]. In both their study (targeting through virus-specific TCRs) and ours (targeting through CAR), lymphoma cells expressing PI9 are sensitive to granzyme-derived killing mechanisms with the Fas-FasL axis only making a minor contribution to cytotoxicity. In a separate study, Kimman et al. explored CD19 and CD20 CAR T killing using a PI9 knockdown lymphoma cell line and demonstrated that at a 1:1 E:T ratio, killing frequencies were similarly not impacted [43]. From a clinical standpoint, PI9 overexpression was significantly associated with better survival rates across patients with nasal T/NK lymphoma [54]. In aggregate, we challenge the idea that PI9 alone is essential for tumor evasion and highlight the importance of redundancy when the primary cytotoxic mechanism, GZMB, is inactivated.

From the viewpoint of cell therapy, our data illustrates that particular subsets of CD8^+^ T cells like T_EM_ express multiple granzymes and hence are likely to be potent effectors that can drive anti-tumor killing. The endodomain of the CAR structure alters the relative frequency of the subsets of CD8^+^ T cells with multiple cytotoxins and hence the anti-tumor potential of the cells that comprise an infusion product. Infusion products of CD19 CAR T cells with the CD28 endodomain are enriched in T cells with multiple granzymes and are primed for immediate killing. In this context, we have recently demonstrated a simple method that takes advantage of the linkage between the migratory potential and cytotoxicity of CD8^+^ T cells to enrich for these cytolytic T cells with high anti-tumor efficacy [24]. Infusion products of CD19 CAR T cells with the 4-1BB endodomain, on the other hand, initially harbor a low frequency of multiple granzyme cytolytic CD8^+^ T cells but however, differentiate into this cytolytic subset after infusion into the patient. Since both approved BCMA CAR T cell products utilize the 41BB endodomain, it likely that they will follow the same trajectory as CD19 CAR T cells yielding the highly cytolytic subset after infusion into the patients [30, 31].

Detailing individual cytotoxic pathways of T cells has been an ongoing process for the past few decades. Numerous studies have highlighted the distinct and significant role of GZMA in inducing cell death by NK and T cells, with the discovery of several downstream mitochondrial and nuclear targets [55–57]. GZMA is characterized as a trypsin-like serine protease that cleaves after basic residues such as lysine and arginine, while GZMB cleaves acidic residues such as aspartic acid (Supplementary Table 2) [58–60]. Although the catalytic efficiency of GZMA is significantly lower than that of GZMB, the association rate constant for GZMA inhibition is 1000-fold lower than the GZMB-PI9 complex (Supplementary Table 1) [45–52]. This difference in cleaving preference and binding affinities highlights the independent functions of these granzymes. Not only is the list of known GZMA substrates expanding, but insights into the different modes of cell death are coming out. Recent studies note that purified GZMA cleaves gasdermin B to induce an inflammatory death known as pyroptosis [61, 62]. With apparent differences in their contributions to cell death, GZMA and GZMB are shown to be structurally and functionally different, further suggesting that GZMA can compensate for dampened GZMB activity in liquid cancers [63].

Solid tumors appear to be more susceptible to extrinsic modes of death such as membrane-bound TNF superfamily proteins [64, 65]. Studies have shown that immune cells tend to switch from granzyme-mediated killing to “slow” death receptor-mediated killing in solid tumor interactions, further supporting our findings [66, 67]. Inhibiting both GZMA and GZMB shows an increase in synaptic and killing times, as T cells likely mediate death via the slower-acting Fas pathway, which significantly contributes to killing by CTLs [63, 68, 69]. We highlight the significance of these pathways relative to each other and the tumor cell [70], implying redundancy in the GZMB pathway of CAR T cells. Unfortunately, not all findings regarding CTLs that get activated through TCRs are necessarily applicable to CAR T cells, thereby emphasizing the need to understand CAR T killing mechanisms.

Herein, our findings demonstrate a deeper understanding of CAR T-mediated killing of tumors and how these cytotoxic pathways can compensate for one another. We demonstrate redundancy in these pathways, indicating that pursuing PI9 inhibition or overexpressing GZMB in immune therapies is not an ideal approach for tumor clearance. Our data suggests that PI9 overexpression by itself is not sufficient to impact killing by CAR T cells, but we recognize that in combination with other immunosuppressive factors such as hypoxia, soluble immunosuppressants (e.g., TGF-β), or PD-L1, and FasL expression on tumor cells can elevate the importance of PI9 as an escape mechanism [71–74]. This knowledge can aid in tackling larger obstacles, such as approaching the tumor microenvironment or systemic responses to CAR T cell therapies. In summary, understanding redundancy in killing mechanisms will allow for the engineering of the next generation of CARs to target the specific mechanisms that allow for tumor escape.

## Materials and methods

### Cell lines

NALM6, Daudi, and Raji cells (ATCC; Manassas, VA) were cultured in RPMI 1640 (Cytiva; Marlborough, MA) and SkOV3 and A375 cells (ATCC) were cultured in DMEM (Corning; Corning, NY). Media was supplemented with 10% fetal bovine serum (FBS) (R&D Systems; Minneapolis, MN), 1% HEPES (Corning), 1% sodium pyruvate (Corning), 1% L-glutamine (Corning), 1% penicillin/streptomycin (Corning). All cell lines were routinely tested for mycoplasma contamination.

### Tumor cell line constructs

SkOV3 and A375 cells were previously transduced with a CD19 surface receptor and sorted via positive CD19 expression [20]. A375-CD19 has 67% CD19 expression (data not shown). For viral particle generation, HEK cells were transfected with lentiviral construct PI9-T2A-mCherry with psPAX2 packaging and pMD2G envelope plasmid DNA. Viral supernatant was collected 48 h later and filtered using a 0.45 µm filter. Tumor cells, SkOV3-CD19 and NALM6 cells were transduced with lentiviral supernatants by centrifugation onto 24-well Retronectin-coated (Takara Bio; Kusatsu, Japan) plates. The tumor cells were expanded and later sorted by mCherry positive expression.

### CAR T cell manufacturing

CAR T cells were manufactured as previously reported [20]. Retroviral particles for CAR T cells were produced by co-transfecting PlatGP cells with CAR and RD114 constructs. The viral particles were filtered using a 0.45 µm filter. PBMCs isolated from whole blood were activated using OKT3 and anti-CD28 antibodies supplemented with IL-7 (10 ng/ml) and IL-15 (5 ng/ml) for 2 days. Activated T cells were transduced with retroviral supernatants by centrifugation onto 24-well Retronectin-coated (Takara) plates, in RPMI media supplemented with IL-7 and IL-15. The cells were allowed to expand for 10 days and the percentage of CAR, CD4, and CD8 T cells was determined by flow cytometry.

### Western blot

Cell lysates were extracted using 1x RIPA buffer (Millipore Sigma; Burlington, MA) and 1x protease inhibitor cocktail (ThermoScientific; Waltham, MA) in deionized water. The protein concentration of the cell lysates were determined using the Pierce BCA Assay Kit (ThermoScientific). The proteins were incubated with 4x Laemmli sample buffer (BioRad; Hercules, CA) and 2-mercaptoethanol (Sigma Life Science, Burlington, MA). The samples were boiled at 95 °C for 5 min and cooled at room temperature for 15 min. 25 µg of the samples were loaded onto a pre-cast 4–15% polyacrylamide gel (BioRad) with 1x tris/glycine/SDS (J.T Baker; Phillipsburg, PA/ VWR Life Science; Radnor, PA/ Hoefer; Holliston, MA) running buffer. The gel ran for 90 min at 100 volts via vertical electrophoresis. The contents from the gel were transferred to a polyvinylidene fluoride (PVDF) membrane (Amersham Hybond; Marlborough, MA) membrane in 1x tris/glycine/methanol (J.T Baker/ VWR Life Science/VWR BDH Chemicals) transfer buffer at 90 volts for 60 min. The PVDF membrane was placed in a blocking buffer of 5% skim milk in 1x tris-buffered saline/TWEEN20 (TBST) (J.T Baker/Fisher Bioreagents; Pittsburgh, PA/SIGMA; St. Louis, MO) buffer for 2 h. The membrane was then incubated overnight with the primary antibody in a solution of 2.5% bovine serum albumin (BSA) (Fisher Bioreagents) in TBST. The primary antibodies used were anti-β-actin antibody (Clone 2F1-1) (Biolegend; San Diego, CA), mouse monoclonal anti-*SERPINB9* antibody (Clone 7D8) (Invitrogen; Waltham, MA), and rabbit monoclonal anti-pro-caspase 3 antibody (Clone D3R6Y) (Cell Signaling Technology; Danvers, MA). The membrane was washed three times for 10 min with TBST then incubated with the secondary antibody in 2.5% BSA in TBST for 60 min. The secondary antibodies used were anti-mouse IgG HRP-linked antibody (CST) and anti-rabbit IgG HRP-conjugated antibody (Jackson ImmunoResearch; West Grove, PA). The membrane was washed three times with TBST and developed using Pierce 1-Step Ultra TMB Blotting Solution (ThermoScientific). Images were taken via cellular device and analyzed using ImageJ.

### Flow cytometry

We phenotyped the CAR T cells using anti-human CD3 (Clone SK7, Biolegend), anti-human CD4 (Clone OKT4, Biolegend), anti-human CD8 (RPA-T8, Biolegend), and specialized CAR antibodies [75]. We used anti-human CD19 (Clone HIB19, Biolegend), anti-human CD95 (Clone DX2, Biolegend), anti-human CD120a (Clone W15099A, Biolegend), and anti-human CD119 (GIR-94, Biolegend) for tumor phenotyping. We used human plasma isolated from whole blood as the blocking solution.

We incubated our cells in FACS buffer (10% FBS in PBS) and human plasma for 30 min at 4 °C. The cells were spun down for 5 min and the desired antibody concentration was added to the cells. The cells and antibodies were co-incubated for 1 h at 4 °C then washed three times with FACS buffer and resuspended in 1 mL of FACS buffer. The samples were then analyzed using the BD LSRFortessa.

For the intracellular staining of PI9, we permealized and fixed our cells using the FOXP3/Transcription Factor Staining Buffer Set (Invitrogen) for 1 h at 4 °C. We incubated our permealized cells with mouse monoclonal anti-*SERPINB9* antibody (Clone 7D8, Biolegend) for 1 h and washed the cells three times. Next, we incubated our cells with anti-mouse IgG (Clone Poly4053, Biolegend) and washed three times. The samples were then analyzed using the BD LSRFortessa.

### Cytotoxicity assay

We coated a 96 well plate with poly-L-lysine (Sigma-Aldrich) for 1 h at 37 °C then subsequently left to dry at room temperature for 30 min. Adherent cells were seeded in the wells for 24 h in DMEM + 10% FBS. The adherent cells were briefly rinsed with phosphate buffer saline (PBS) (Corning) and labeled with BioTracker 490 Green Cytoplasmic Membrane Dye (Sigma-Aldrich) for 30 min, then rinsed with complete media. Suspension cells were washed with PBS at 350 g for 5 min and labeled with BioTracker 490 for 30 min. The cells were washed in full media were seeded into wells pre-coated with poly-L-lysine. Next, the effector cells were washed with PBS and loaded into the respective wells. The co-cultures were in a 200 µl solution of IMDM (Gibco; Billings, MT), 10% FBS and Annexin V Alexa Fluor 647 (Invitrogen). The plate was imaged using BrightField, GFP, and CY5 filters for 48 h every 3 h at 37 °C and 5% CO_2_ via Cytation (BioTek; Winooski, VT). The images were extracted and analyzed via ImageJ.

### Inhibition/blocking cytotoxicity assay

The cytotoxicity assay procedure was prepared as mentioned previously. For GZMB inhibition, the effector cells were pre-incubated in IMDM + 10% FBS supplemented with 100 µM Z-AAD-CMK (Enzo Life Sciences; Farmingdale, NY) for 1 h. The effector cells were then added to the wells in a working solution of IMDM,10% FBS and 100 µM Z-AAD-CMK. For GZMA inhibition, the effector cells were pre-incubated in IMDM + 10% FBS supplemented with 5 µM nafamostat mesylate (Selleck Chem; Houston, TX) for 1 h. The effector cells were then added to the wells in a working solution of IMDM,10% FBS and 5 µM nafamostat mesylate.

For antibody blocking, the effector cells were incubated with 10 µg/mL anti-human FasL (CD178) monoclonal antibody (Clone NOK1, BioLegend) for 1.5 h with periodic agitation. The effector cells were then added to the wells in a working solution of IMDM,10% FBS and 10 µg/mL anti-FasL antibody. For IFN-γ and TNF-α blocking, the effector cells were incubated with 5 µg/mL anti-human IFN-γ monoclonal antibody (Clone 4 S.B3, BioLegend) and 5 µg/mL anti-human TNF-α monoclonal antibody (Cline MT25C5, Mabtech; Cincinnati, OH) for 1.5 h with periodic agitation. The effector cells were then added to the wells in a working solution of IMDM,10% FBS and 5 µg/mL anti-IFN-γ and anti-TNF-α antibodies.

### TIMING assays

Nanowell arrays on a chip were fabricated as previously reported [76]. The chip was placed in a plasma chamber (Harrick Plasma Inc; Ithaca, NY) for two min and treated with PLL[20]-g[3.5]- PEG(2)/PEG(3.4)- biotin(50%) (SuSoS; Zurich, Switzerland) for assays using SkOV3. Target and effector cells were washed three times with PBS prior to staining. We labeled the target and effector cells with PKH26 Red and PKH67 Green (Sigma-Aldrich), respectively. Cells were then washed three times in full media and resuspended at a density of 1 million cells/mL. Effector and target cells were loaded onto the nanowell arrays and resuspended in IMDM, 10% FBS and Annexin V Alexa Fluor 647. The chip was imaged in BrightField, Alexa Fluor 488, TexasRed, and Cy5 using a Zeiss Axio Observer (Oberkochen, Germany) for 6 h (suspension cells) or 7 h (adhesive cells) over 73 time points. Image analysis and cell tracking were performed as previously reported [76].

### CD8 staining

Following the TIMING assay, we blocked the chip with human plasma isolated from whole blood. We incubated the chip with anti-human CD8 (Clone RPA-T8, Biolegend) on ice for 1 h and imaged the chip using the DAPI filter on the Zeiss Axio Observer. Events were binarized via a fluorescence threshold set at 125 a.u.

### Design and transduction of fluorescent reporters

The design of the reporters was modeled using the Bardet design [77]. The fluorescent reporter is composed of a signal peptide LLLVTSLLLCELPHPAFLLIP, Myc tag, GS spacer, human CD8 transmembrane domain, DIAP1 domain with three consecutive VGPD’SG protease sites, SV40 nuclear localization signal, and enhanced green fluorescent protein (eGFP). The design was finalized using SnapGene.

The plasmid backbone was propagated using electrochemically *E. cloni* competent cells and isolated using a mini-prep kit (Qiagen; Hilden, Germany). We amplified the plasmid via Q5 DNA polymerase (New England Biolabs; Ipswich, MA) and treated it with restriction enzymes BsiWI (NEBiolabs) and EcoRI (NEBiolabs) overnight. The plasmid was purified via 0.6% agarose gel electrophoresis for 90 min at 75 V. The plasmid (60 ng/µl) was combined with our gene insert and ligated via Gibson assembly (NEBiolabs). Electrocompetent cells were transformed with the Gibson products and the samples were amplified via colony PCR using *Taq* polymerase (NEBiolabs) with an extension time of 1.5 min. The colony PCR products were separated via 1% agarose gel for 90 min at 75 V. Positive colonies were submitted for Sanger sequencing.

The reporter plasmid was transfected into HEK293 metr cells with *pMd2G* and *psPAX2* helper plasmids using Lipofectamine LTX (Invitrogen). The cells were incubated with the plasmids for 72 h and the supernatant was collected and filtered using a 0.2 µm filter. The GZMB reporter plasmid was virally transduced into NALM6 and NALM6-PI9 and expanded for a week. The transduced cells were sorted based on GFP expression.

### Fluorescent reporter assays

We incubated 1000 U of activated GZMB (Enzo Life Sciences) and 0.5 mg/mL perforin (RayBiotech; Norcross, GA) with NALM6-GBR. After 24 h, we stained the cells with DRAQ5 nuclear stain (Novus Biologicals; Centennial, CO) and imaged the cells using a Nikon confocal microscope (Tokyo, Japan). We used ImageJ to measure the Pearson correlation coefficient of the nuclear stain and reporter. The reporter cells were co-cultured with CAR T cells in the nanowell chip for TIMING. Translocation frequencies were determined for cell death events and validated using the nuclear detection module.

### Nuclear translocation through label-free nucleus detection

We quantified the signal translocation of the reporter against cell nuclei through automated nucleus detection of the cells [78]. We used the Mask-RCNN model adapted previously for label-free nucleus detection. We further validated the model using the TIMING dataset with DAPI staining as ground truth for the nucleus mask containing 1 123 Jurkat cells and 1 188 NALM6 cells. We performed model validation following the procedure similar to previous work: first, we generated binary masks using the blob detection based on the Laplacian of Gaussian filter, the flood fill algorithm, and the closing operation [78]. With binary masks as ground truth, we then calculated the intersection over union (IoU) accuracy of model predictions against ground truth masks and the Pearson correlation coefficient (PCC) value of prediction against the original fluorescent signal. These two matrices will indicate how well the predicted masks overlap against ground truth in the form of binary masks and the original 8-bit signal.

After model validation, we combined the cell detection module in the TIMING pipeline and the nucleus detection model to assign nucleus masks to individual cells [79]. For each cell, the cell detection model will define a bounding box. To quantify signal translocation, we calculate the PCC value of the binary nuclear mask against the 8-bit signal from the reporter channel within the bounding box. A high PCC value indicates pixels with a stronger signal distributed within the nuclear mask. At random, we picked 24 images for cells with and without a nuclear translocation to quantify signal translocation.

### Anti-Fas TIMING assays

Effector cells were washed three times with PBS, stained with PKH67 and subsequently washed with full media. The effector cells were incubated with 10 µg/mL anti-human FasL (CD178) monoclonal antibody (Clone NOK1, BioLegend) for 1.5 h with periodic agitation. The target cells were washed and stained using the previous methodology. The cells were loaded onto the nanogrid array and resuspended in IMDM, 10% FBS, Annexin V Alexa Fluor 647, and 10 µg/mL Anti-FasL. The chip was imaged for 6/7 h depending on the target cell line. Image analysis and cell tracking were performed as previously described.

### GZMA inhibitor TIMING assays

Effector cells were washed three times with PBS, stained with PKH67 and subsequently washed with full media. The effector cells were incubated with 5 µM nafamostat mesylate (Selleck Chem; Houston, TX) for 1.5 h with periodic agitation. The target cells were washed and stained using the previous methodology. The cells were loaded onto the nanogrid array and resuspended in IMDM, 10% FBS and Annexin V Alexa Fluor 647 and 5 µM nafamostat mesylate. The chip was imaged for 6/7 h depending on the target cell. Image analysis and cell tracking were performed as previously mentioned.

### scRNAseq analysis

Single-cell RNA-seq datasets were accessed under GEO accession GSE197268 and GSE208052 for Tisa-cel and Axi-cel infusion products [23, 24]. We used the Seurat package in R and SAVER for expression recovery of the samples. Quality control was performed by removing cells with feature counts over 6 500 and below 150, and mitochondrial counts over 15%. We normalized the data using a log transformation and scaled the data. We dimensionally reduced the cells via PCA and UMAP. The single cells were filtered for positive CD3 and CAR expression and separated based on CD4/CD8 expression.

### Statistical analysis

Time values within the text are written as the mean ± standard deviation. Statistical testing was performed using GraphPad Prism (v8.0). Cytotoxicity plots show the mean percentage of three biological replicate wells (N = 3) and the error bars show the SEM. Statistical testing for the cytotoxicity assays was performed at each time point using multiple Mann-Whitney tests. For bar graphs, statistical testing was performed using the chi-square test of independence. For violin plots, statistical testing was performed using the unpaired t-test when n > 25 and the Mann-Whitney test when n < 25. TIMING experiments were repeated to reach a minimum sample size of 20 events. Data exclusion was applied to the TIMING experiments: if the tumor and immune cell were interacting at t = 0, the well was excluded from kinetic analysis. Schematics were generated using Microsoft Powerpoint and Biorender via license.

### Supplementary information

Supplementary Figures

Supplementary Table 1

Supplementary Table 2

Supplementary Movie 1

Supplementary Movie 2

Supplementary Movie 3

Supplementary Movie 4

Original Western blots

## Data Availability

The datasets analyzed within the current study are available in the GEO repository, under accession numbers GSE197268, GSE208052, and GSE223655 [23, 24, 80].

## Code availability

All relevant package and software information is provided in the methods section. We have no novel code to report for this study.
