# Supplementary material for: Decoding the mechanisms of chimeric antigen receptor (CAR) T cell-mediated killing of tumors: insights from granzyme and Fas inhibition
Source: Cell Death Dis. 2024 Feb 2;15(2):109. doi: 10.1038/s41419-024-06461-8 (PMC10837176; doi:10.1038/s41419-024-06461-8)
Supplement: Supplementary file 1 — Supplementary Figures [file 41419_2024_6461_MOESM1_ESM.docx]

**Supplementary Figures**

**Decoding the mechanisms of Chimeric Antigen Receptor (CAR) T cell-mediated killing of tumors: insights from granzyme and Fas inhibition**

Melisa J. Montalvo, Irfan N. Bandey, Ali Rezvan, Kwan-Ling Wu, Arash Saeedi, Rohan Kulkarni, Yongshuai Li, Xingyue An, Samiur Rahman Sefat, and Navin Varadarajan

**
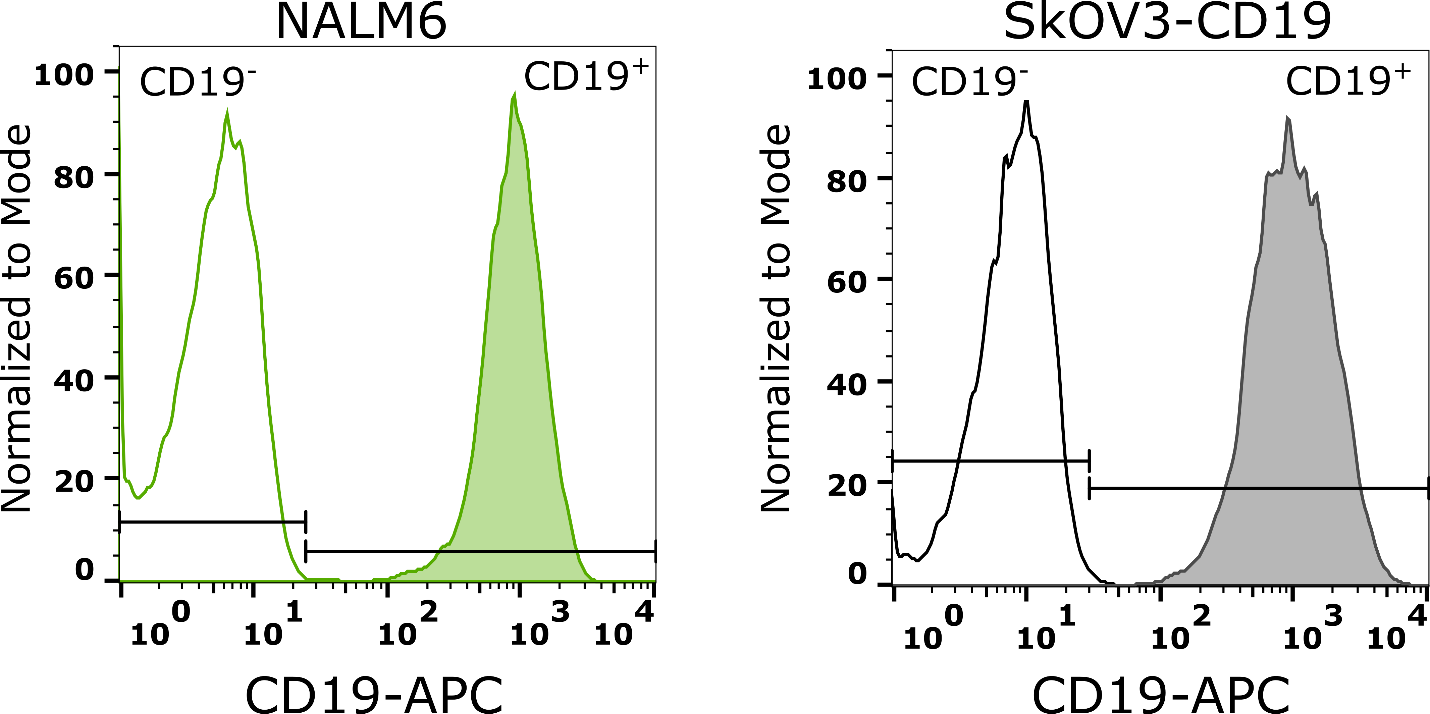
Figure S1. Positive CD19 surface expression of NALM6 and SkOV3-CD19 via flow cytometry.** NALM6/SkOV3-CD19 cells stained with CD19-APC are shown as the filled curve and unstained cells represent the negative control (empty curve).

**
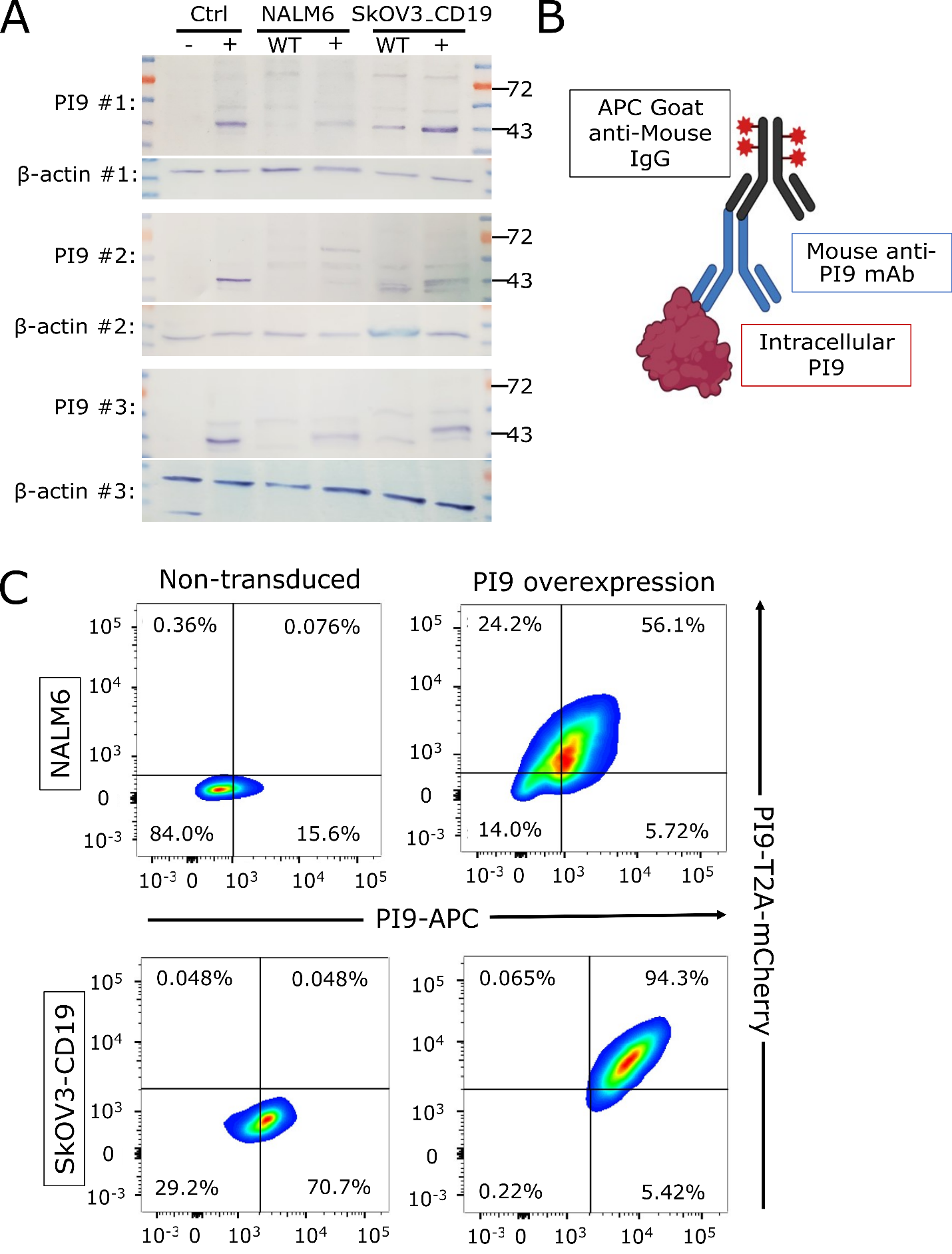
**

**Figure S2. Validation of PI9 overexpression in NALM6 and SkOV3-CD19.**
(A) Western blot biological replicates for PI9 and β-actin expression of target cell
 lines. Original Western blots attached as supplementary image file.
(B) Schematic of indirect flow cytometry detection of PI9.
(C) Flow cytometry panel for indirect detection of PI9. Left two panels are wild-type
 NALM6 and SkOV3-CD19 and right two panels are PI9 overexpressing cells. X-
 axis shows PI9-APC detection and y-axis shows mCherry detection of PI9-T2A-
 mCherry vector construct.


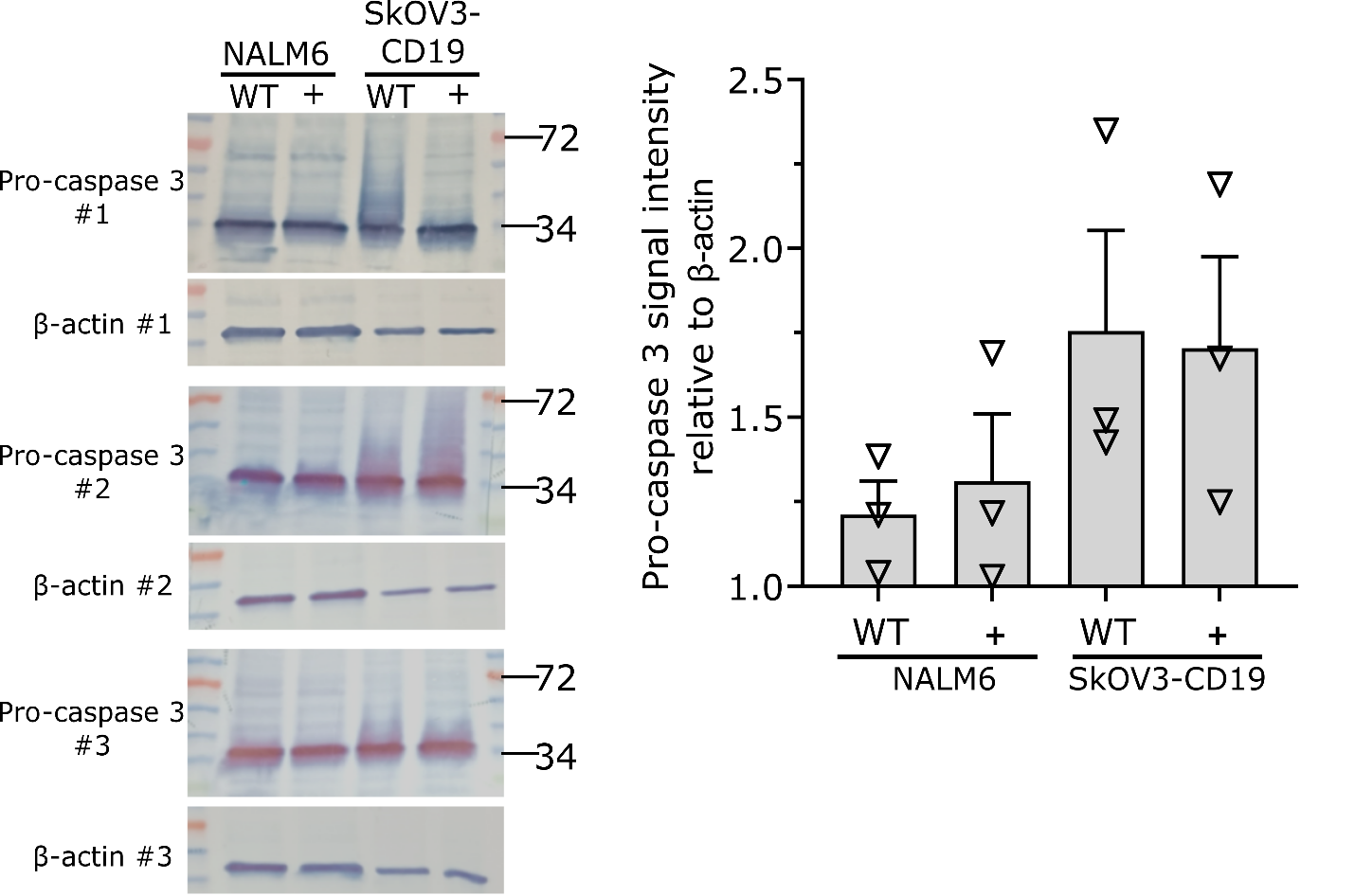


**Figure S3. Western blot results of pro-caspase 3 expression in cell lysates overexpressing PI9 compared with wild-type cells.** The bar graph represents the mean gray value of the pro-caspase 3 bands normalized in relation to the loading control β-actin. Pro-caspase 3 has a predicted molecular weight of 32 kDa. Original Western blots attached as supplementary image file.

**
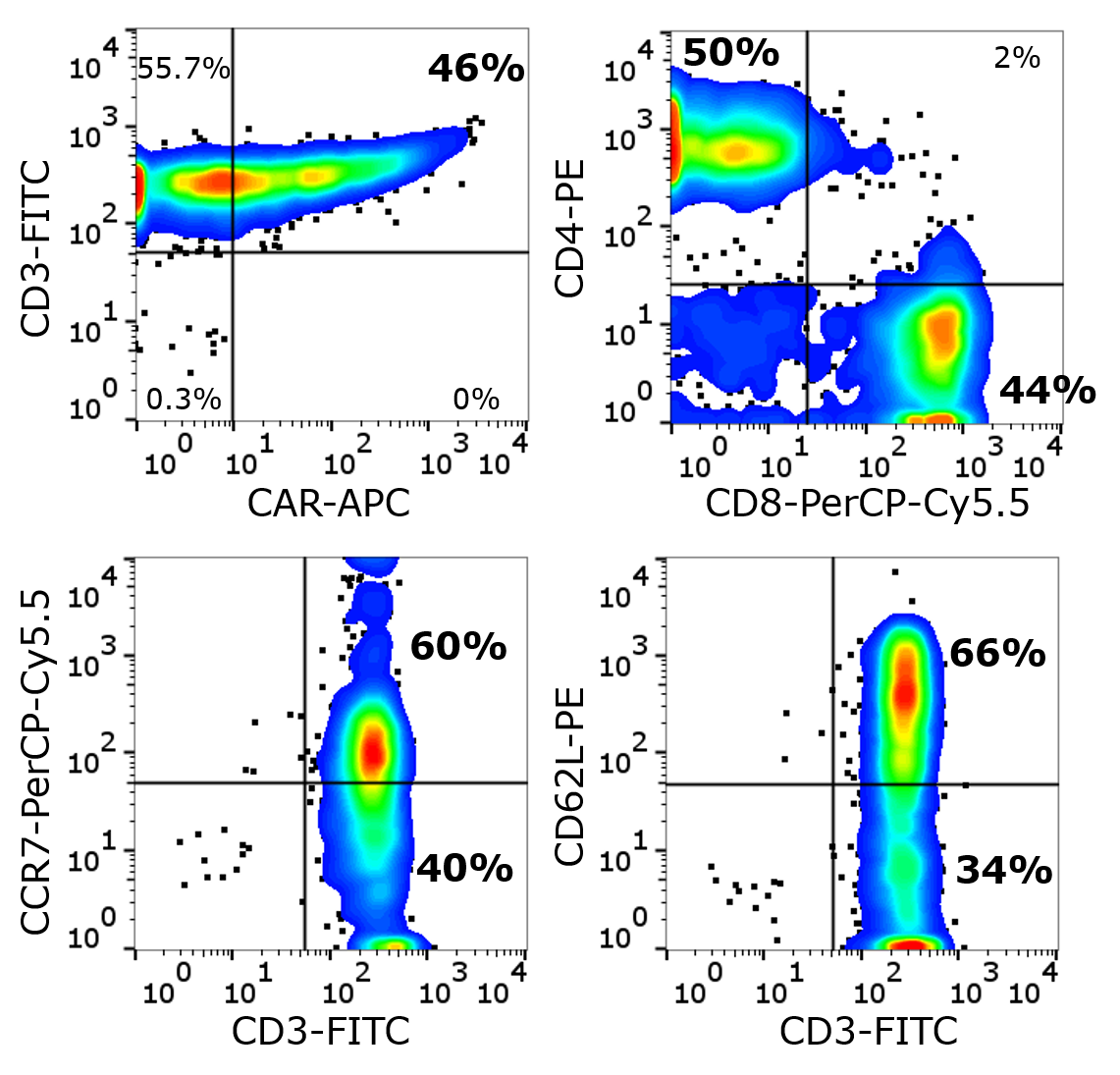
**

**Figure S4. Representative flow phenotyping of 19-41BB CAR T cells from one donor.** The second donor (not shown) expressed 64% CAR and 30%:60% CD8:CD4.


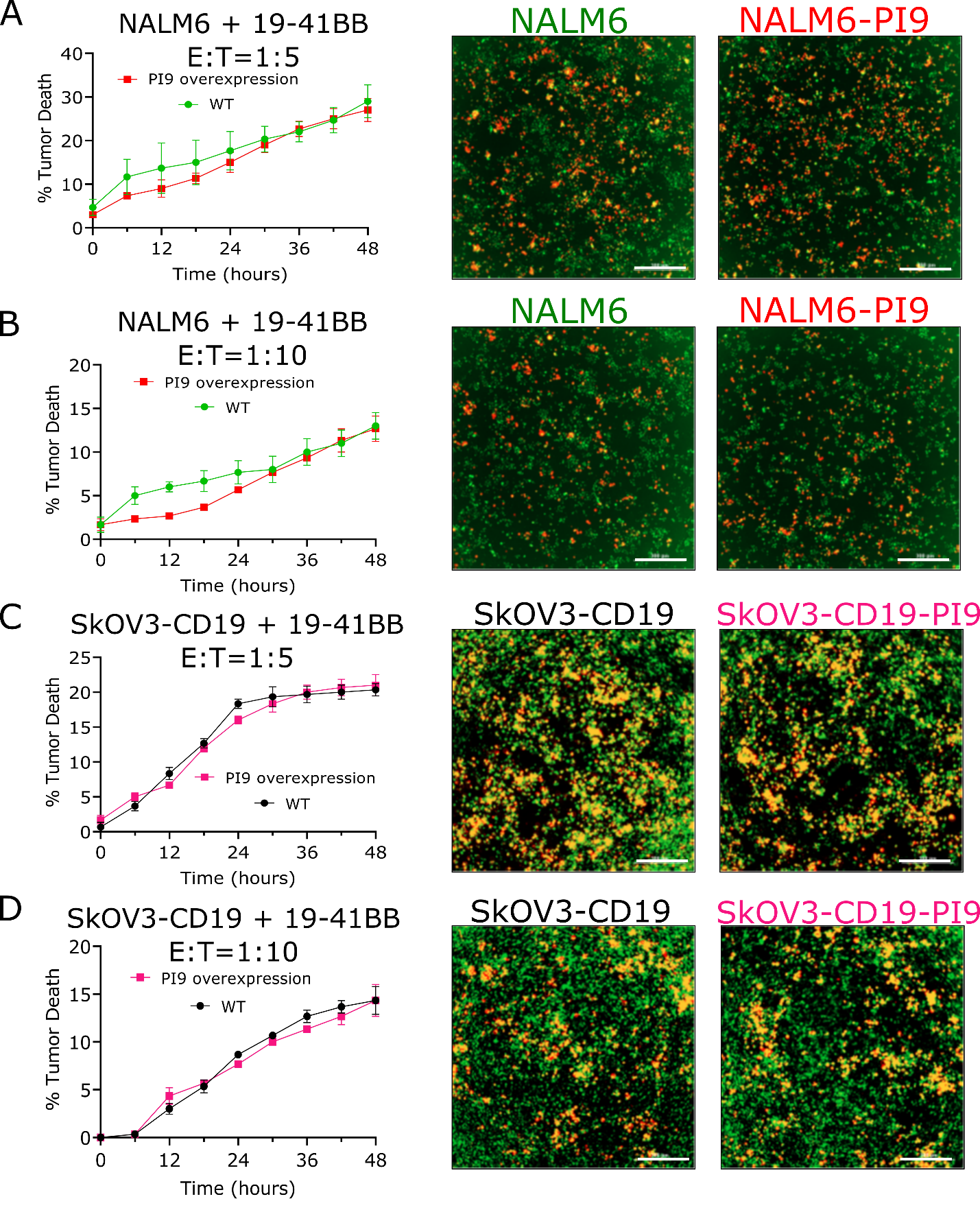


**Figure S5. PI9 overexpression does not significantly impact 19-41BB CAR T killing at higher effector-to-target ratios in cytotoxicity assays.**

(A/B) Time-dependent cytotoxicity of 19-41BB CAR T cells against NALM6 and NALM6-PI9 at an effector-target ratio of 1:5 and 1:10, respectively. Cytotoxicity percentage was corrected by subtracting the spontaneous target death percentage via tumor only control wells. The tumor cells are green, the death marker is red and dead tumors appear yellow. The scale bar represents 300 µm.

(C/D) Time-dependent cytotoxicity plot of 19-41BB CAR T cells against SkOV3-CD19 and SkOV3-CD19-PI9 at an effector-target ratio of 1:5 and 1:10, respectively. Cytotoxicity percentage was corrected by subtracting the spontaneous target death percentage via tumor only control wells. The tumor cells are green, the death marker is red and dead tumors appear yellow. The scale bar represents 300 µm.

* Cytotoxicity plots show the mean percentage of three replicate wells (N=3) and the error bars show the SEM. Statistical testing for the cytotoxicity assay was performed at each time point using multiple Mann-Whitney tests.


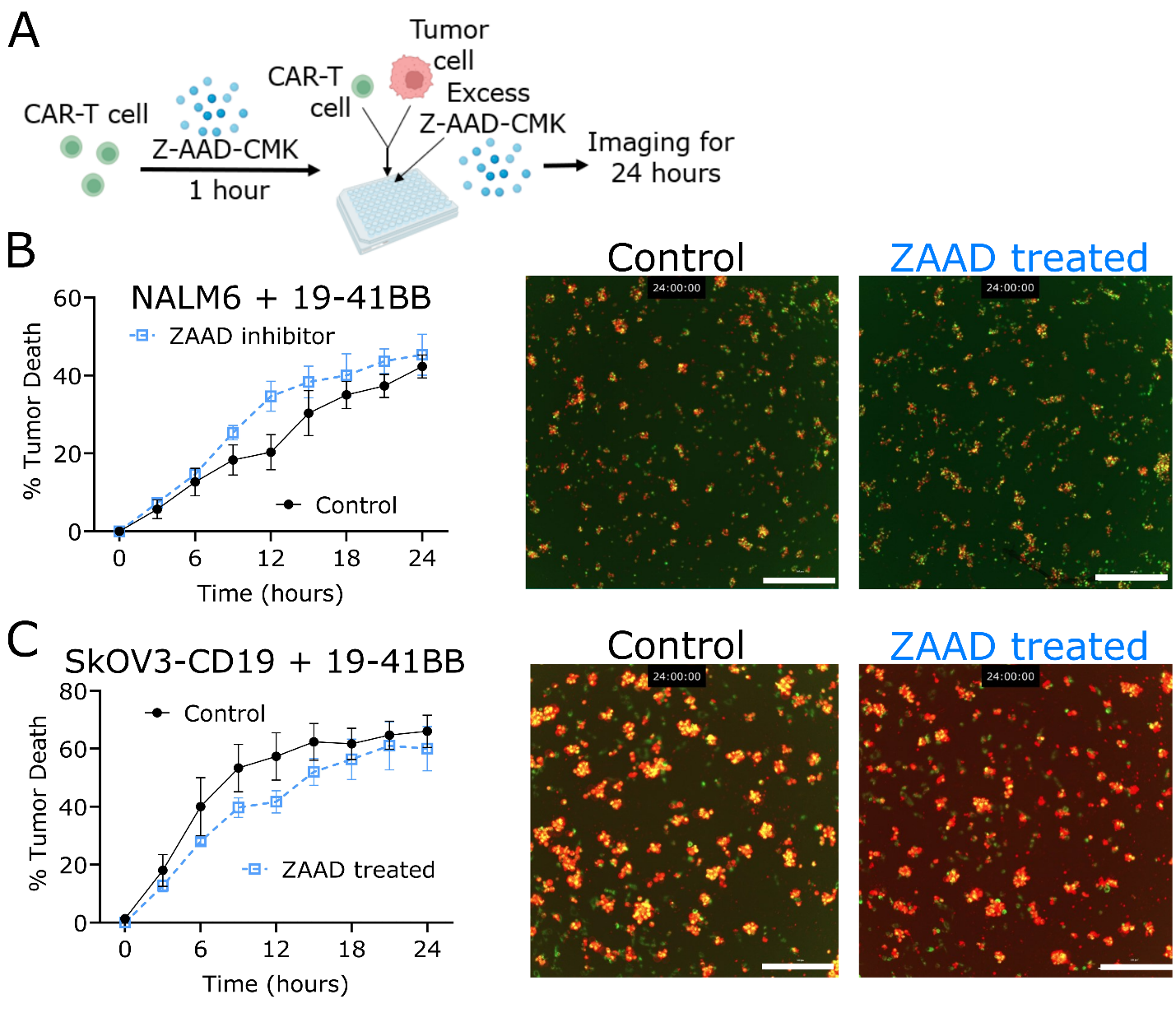


**Figure S6. Inhibition with Z-AAD-CMK does not significantly impair 19-41BB CAR T killing in cytotoxicity assays.**

1. Schematic of cytotoxicity assay with synthetic inhibition of granzyme B.
2. Time-dependent cytotoxicity plot of 19-41BB CAR T cells against NALM6 with
    and without Z-AAD-CMK treatment. Cytotoxicity percentage was corrected by
    subtracting the spontaneous target death percentage via tumor only control
    wells. Microscopy images at 24 hours were extracted from one of the replicate
    wells. The tumor cells are green, the death marker is red and dead tumors appear
    yellow. The scale bar represents 300 µm.
3. Time-dependent cytotoxicity plot of 19-41BB CAR T cells against SkOV3-CD19
    with and without Z-AAD-CMK treatment. Cytotoxicity percentage was corrected
    by subtracting the spontaneous target death percentage via tumor only control
    wells. Microscopy images at 24 hours were extracted from one of the replicate
    wells. The tumor cells are green, the death marker is red and dead tumors appear
    yellow. The scale bar represents 300 µm.

   * Cytotoxicity plots show the mean percentage of three replicate wells (N=3) and the error bars show the SEM. Statistical testing for the cytotoxicity assay was performed at each time point using multiple Mann-Whitney tests.


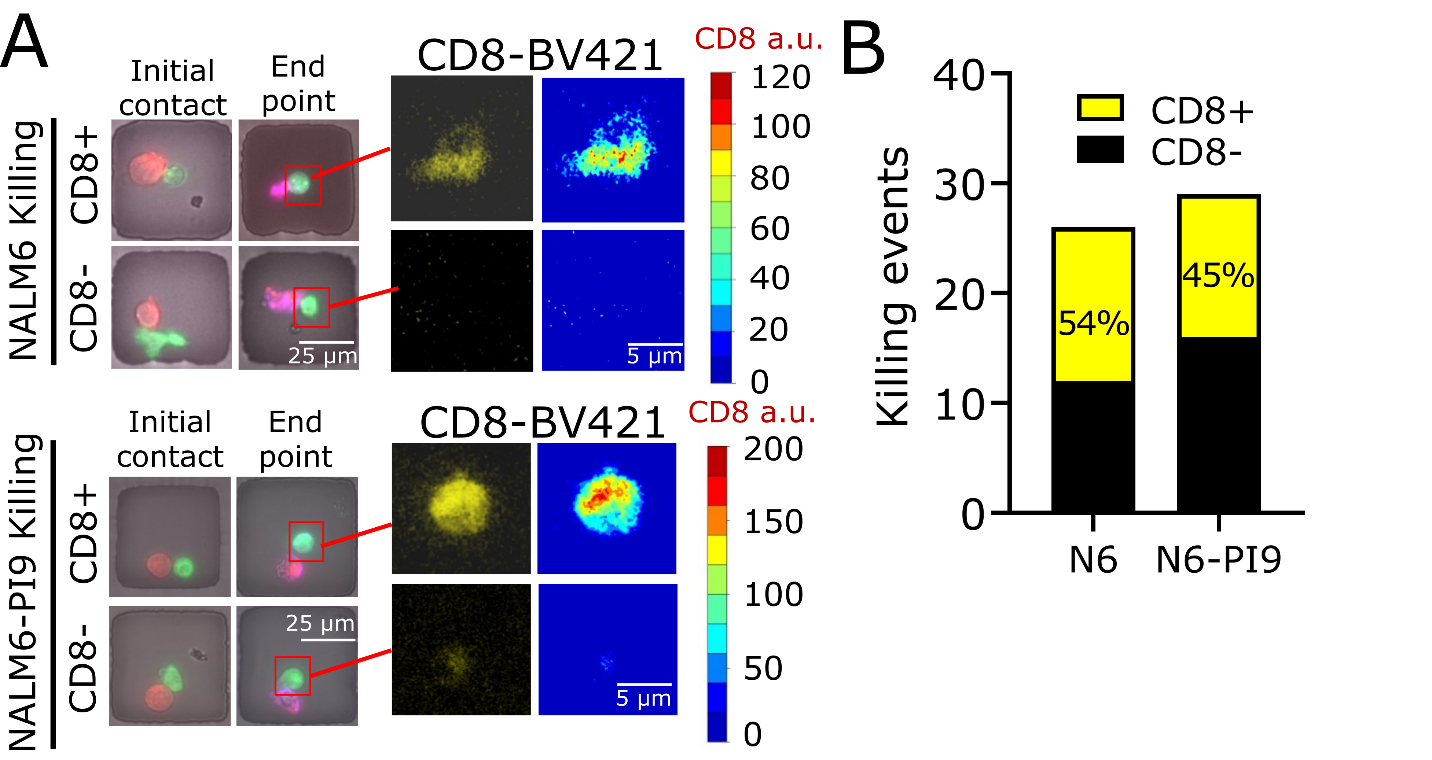


**Figure S7. Killing events are not exclusive to CD8^+^ CAR T cells.**

1. Micrographs showing CD8-BV421 staining intensity of killer T cells against NALM6 and NALM6-PI9. First frame shows the point of initial T cell to tumor conjugation and the 2^nd^ frame shows the end point microscopy image after CD8 staining and tumor death. The 3^rd^ frame is the DAPI filter channel to show CD8-BV421 staining then transformed into a filled contour plot in the 4^th^ frame.
2. Bar graph showing percentage of killing events by CD8^+^ and CD8^-^ T cells.

*For the bar graph, statistical testing was performed using the Chi-square test of independence.


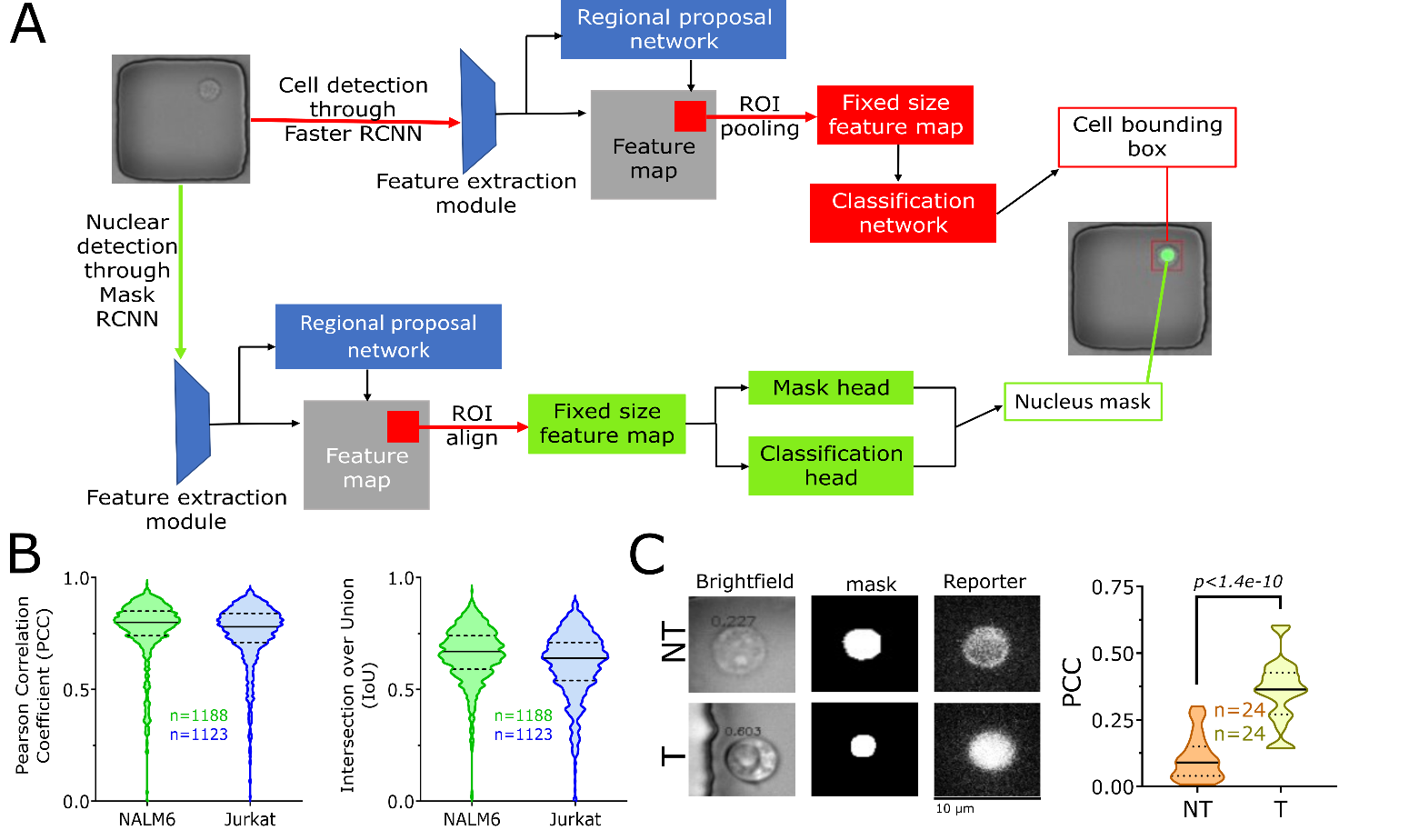

**Figure S8. Automated detection of the localization of fluorescent reporters to the nucleus of cell lines.**
(A) Overview of nuclear detection module.
(B) Violin plots for model validation of NALM6 and Jurkat cell training. The PCC is
 the model prediction against the original fluorescence intensity and the IoU
 shows the accuracy of the model prediction against the ground truth mask.
(C) Microscopy images of nuclear mask validation with granzyme B reporter. Violin
 plot of the overlay Pearson correlation coefficient between nuclear mask and
 reporter of non-translocated and translocated events.

*For the violin plots, statistical testing was performed using the unpaired t-test. The solid line represents the median and the dotted lines represent the upper and lower quartiles. Asterisks denote the following significant p-values: *p < 0.05, **p < 0.01. ***p < 0.001, ****p < 0.0001.


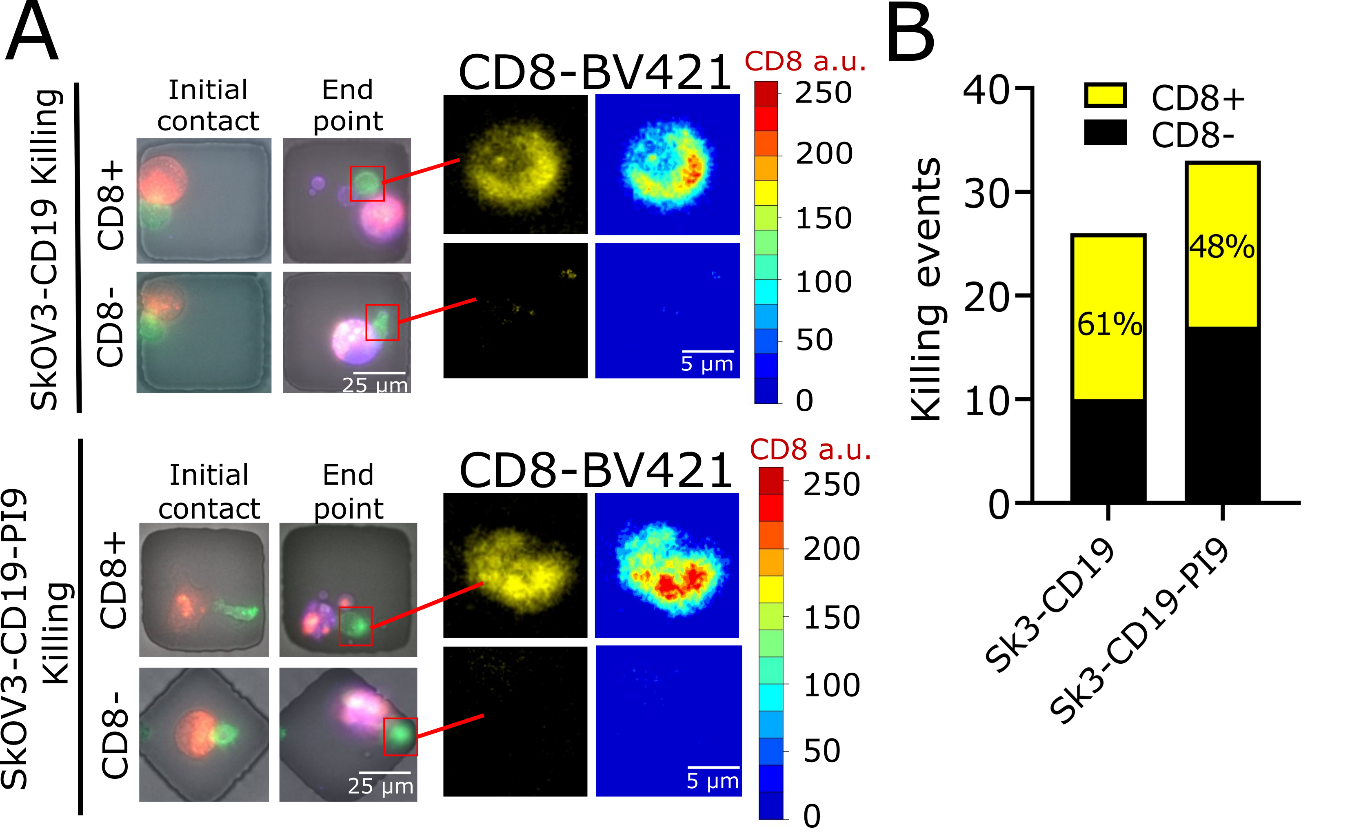


**Figure S9. SkOV3 killing events are not exclusive to CD8+ CAR T cells.**

1. Micrographs showing CD8-BV421 staining intensity of killer T cells against SkOV3-CD19 and SkOV3-CD19-PI9. First frame shows the point of initial T cell to tumor conjugation and the 2^nd^ frame shows the end point microscopy image after CD8 staining and tumor death. The 3^rd^ frame is the DAPI filter channel to show CD8-BV421 staining then transformed into a filled contour plot in the 4^th^ frame.
2. Bar graph showing percentage of killing events by CD8^+^ and CD8^-^ T cells.

*For the bar graph, statistical testing was performed using the Chi-square test of independence.

**
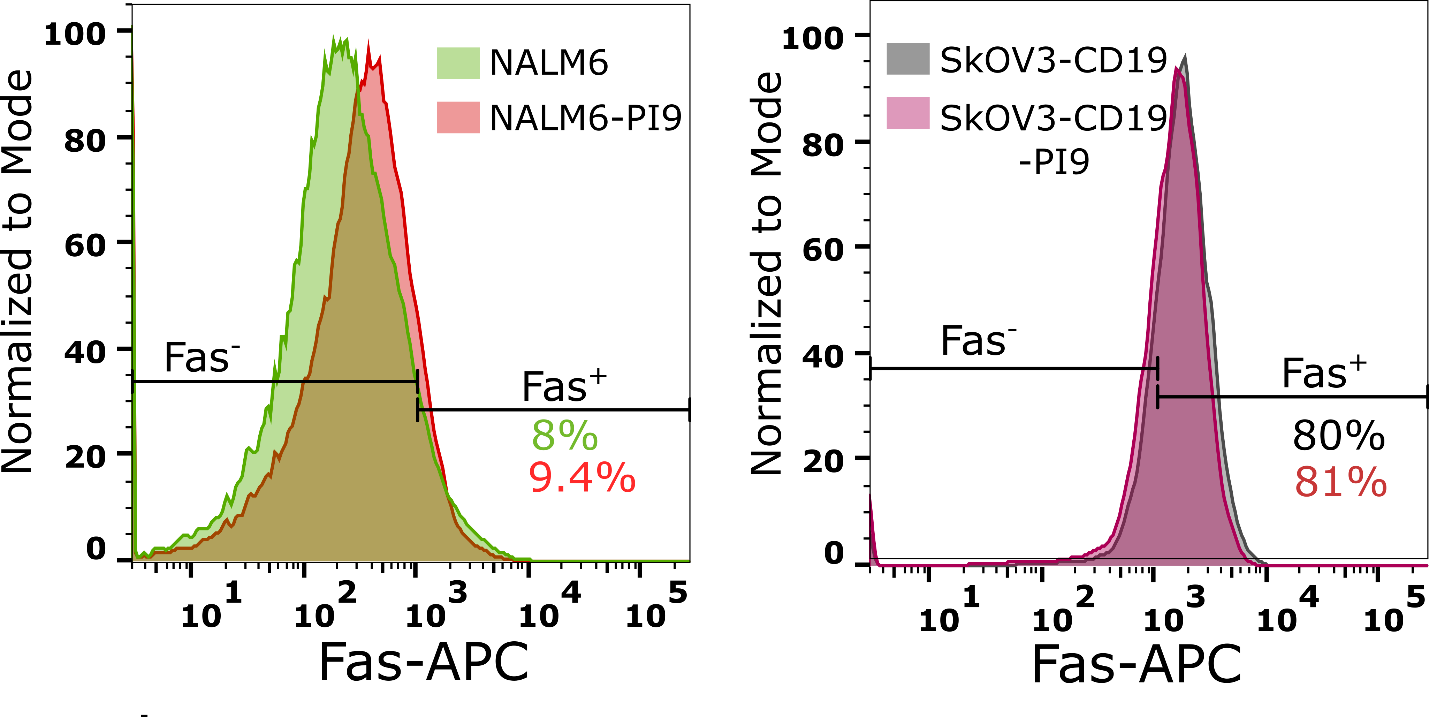
**

**Figure S10. Flow cytometry shows Fas expression is not affected by changes in PI9 expression.** Normalized histograms (left) showing Fas-APC expression in NALM6 (green) and NALM6-PI9 (red) and normalized histograms (right) showing Fas-APC expression in SkOV3-CD19 (gray) and SkOV3-CD19-PI9 (pink).


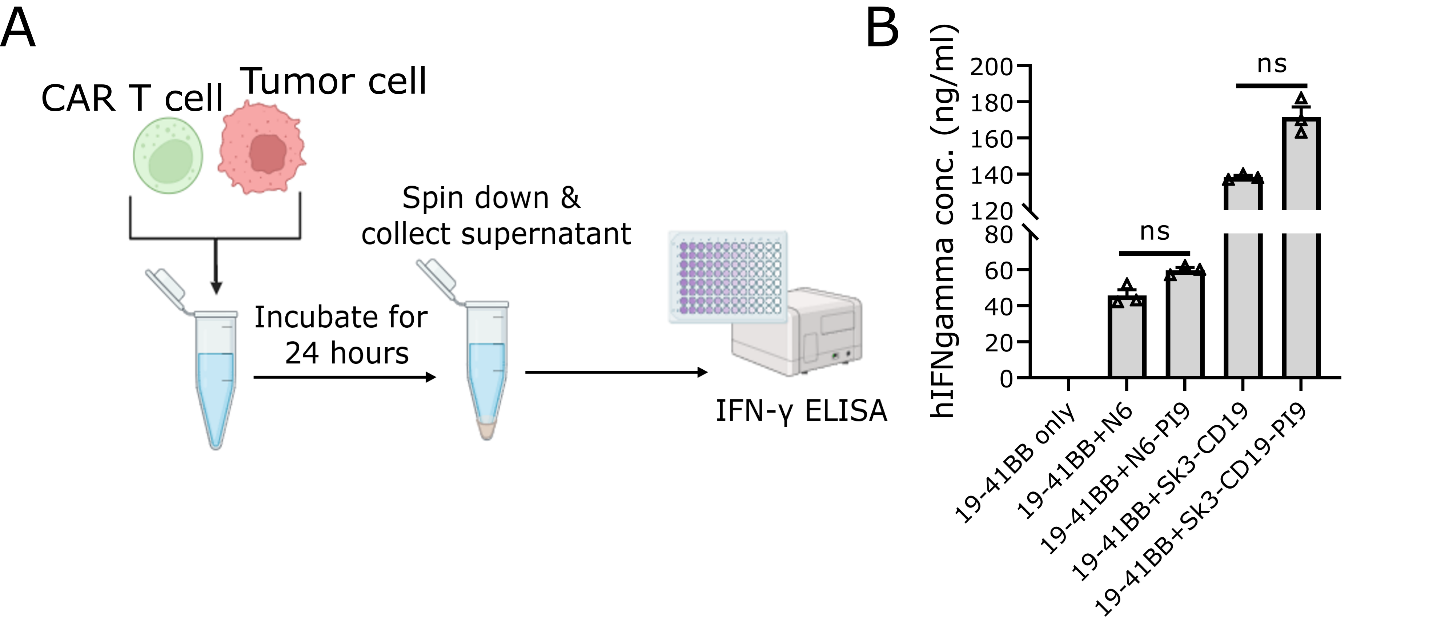


**Figure S11. 19-41BB CAR T cells release IFN-γ by interacting with tumor cells.**

1. Overview of the experimental design for assaying IFN-γ secreted by T cells upon activation by tumor cells.
2. ELISA based quantification of human IFN-γ secreted by 19-41BB CAR T cells co-incubated with and without tumor cells. Bar graphs show the mean from three biological replicates. Error bars show the SEM.


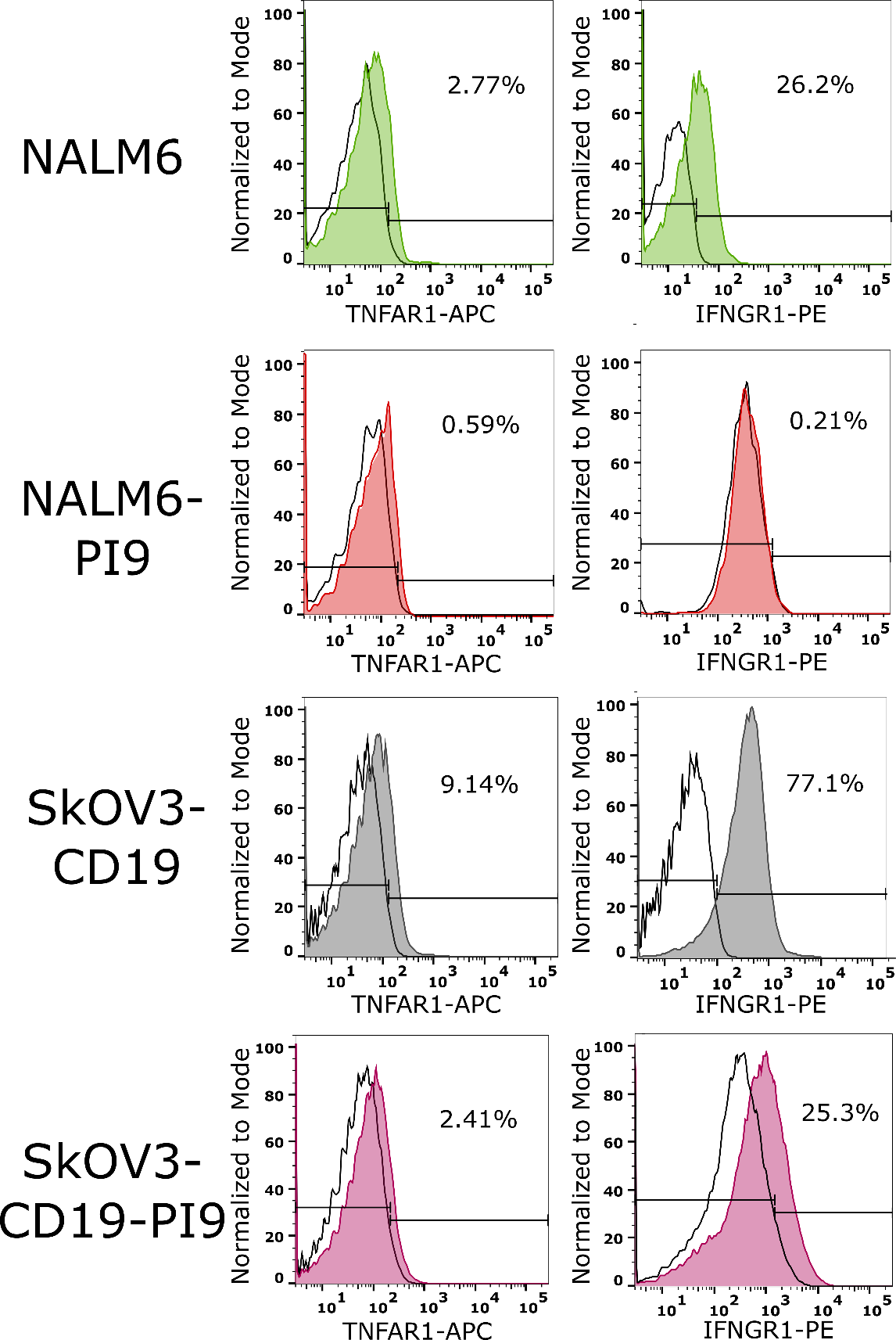


**Figure S12. PI9 overexpressing cells show lower frequencies of IFN-γ and TNF-a receptors.** The colored curves represent the cell staining and the black curves represent the negative control per cell line.

**
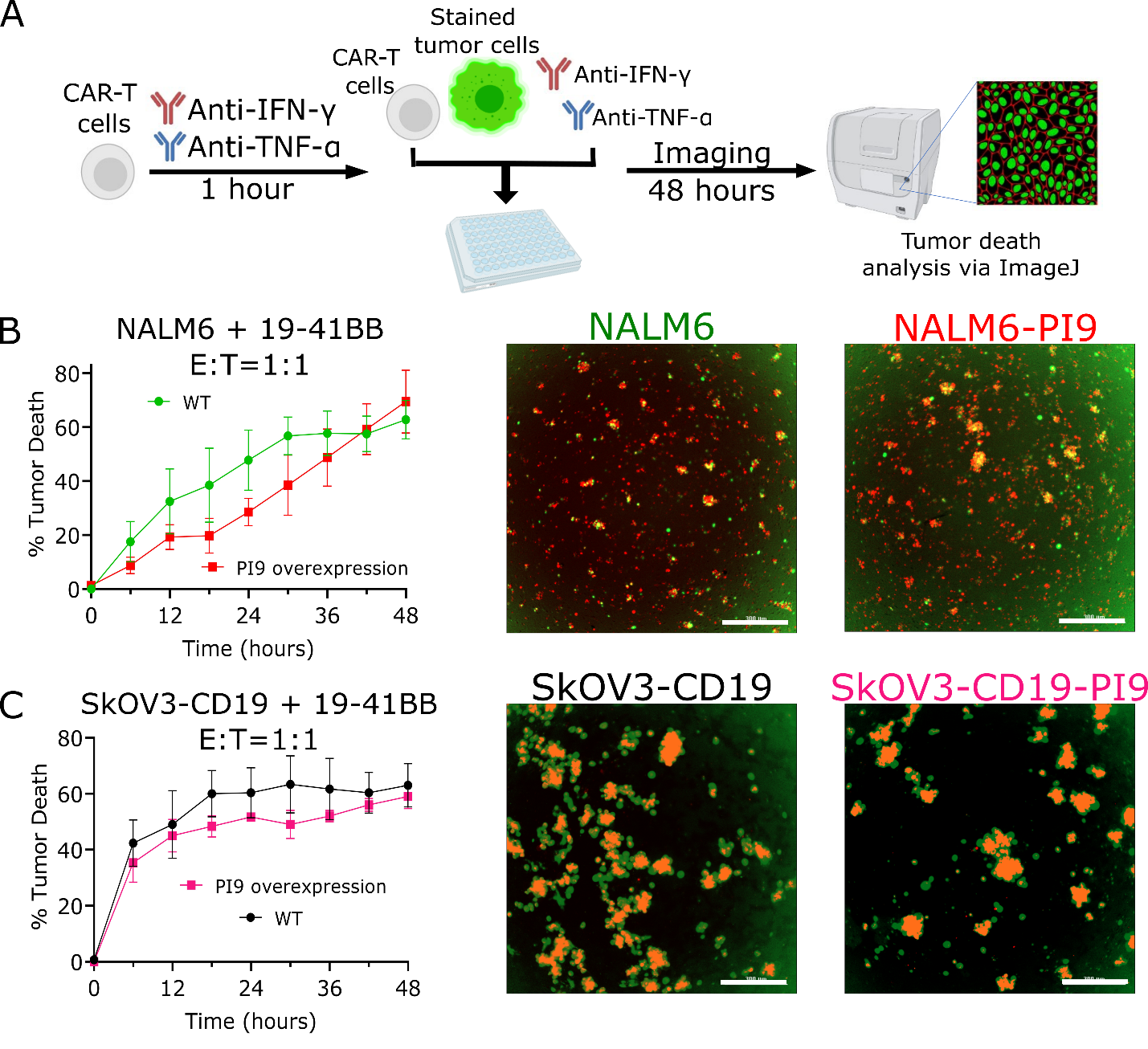

Figure S13. Overexpressing PI9 and blocking IFN-γ and TNF-α secretion does not impact killing by 19-41BB CAR T cells in cytotoxicity assays.**

1. Schematic of cytotoxicity assay using monoclonal anti-IFN-γ and anti-TNF-α antibodies to block interaction between the cytokine and tumor cell targets.
2. Time-dependent cytotoxicity plot of 19-41BB T cells against NALM6 and NALM6PI9 with anti-IFN-γ and anti-TNF-α antibodies. Cytotoxicity percentage was corrected by subtracting the spontaneous target death percentage via tumor only control wells. The tumor cells are green, the death marker is red and dead tumors appear yellow. The scale bar represents 300 µm.
3. Time-dependent cytotoxicity plot of 19-41BB T cells against NALM6 and NALM6PI9 with anti-IFN-γ and anti-TNF-α antibodies. Cytotoxicity percentage was corrected by subtracting the spontaneous target death percentage via tumor only control wells. The tumor cells are green, the death marker is red and dead tumors appear yellow. The scale bar represents 300 µm.

* Cytotoxicity plots show the mean percentage of three replicate wells (N=3) and the error bars show the SEM. Statistical testing for the cytotoxicity assay was performed at each time point using multiple Mann-Whitney tests.


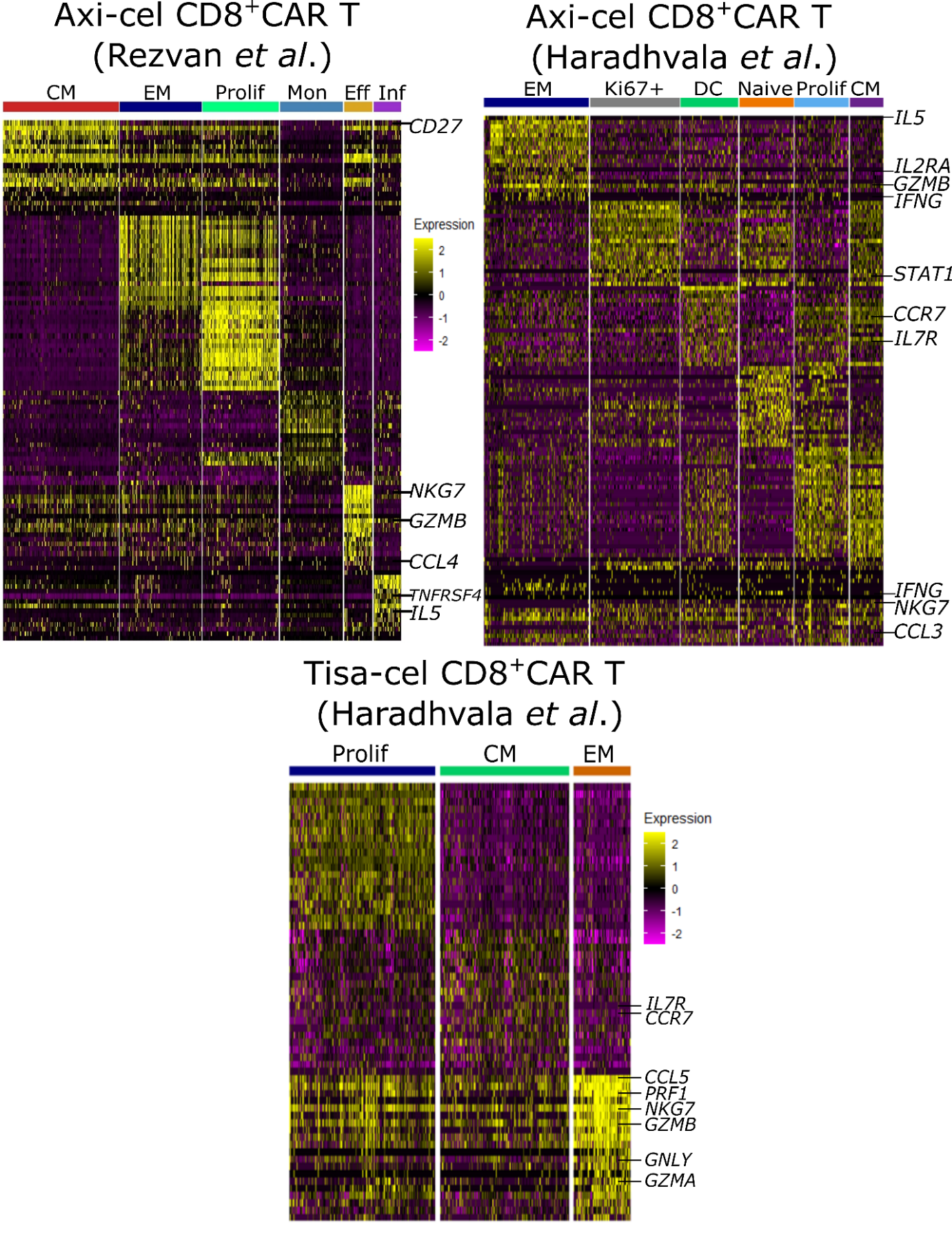


**Figure S14. Heatmaps of clustered infusion products show upregulation of cytotoxic genes distributed across all clusters.**


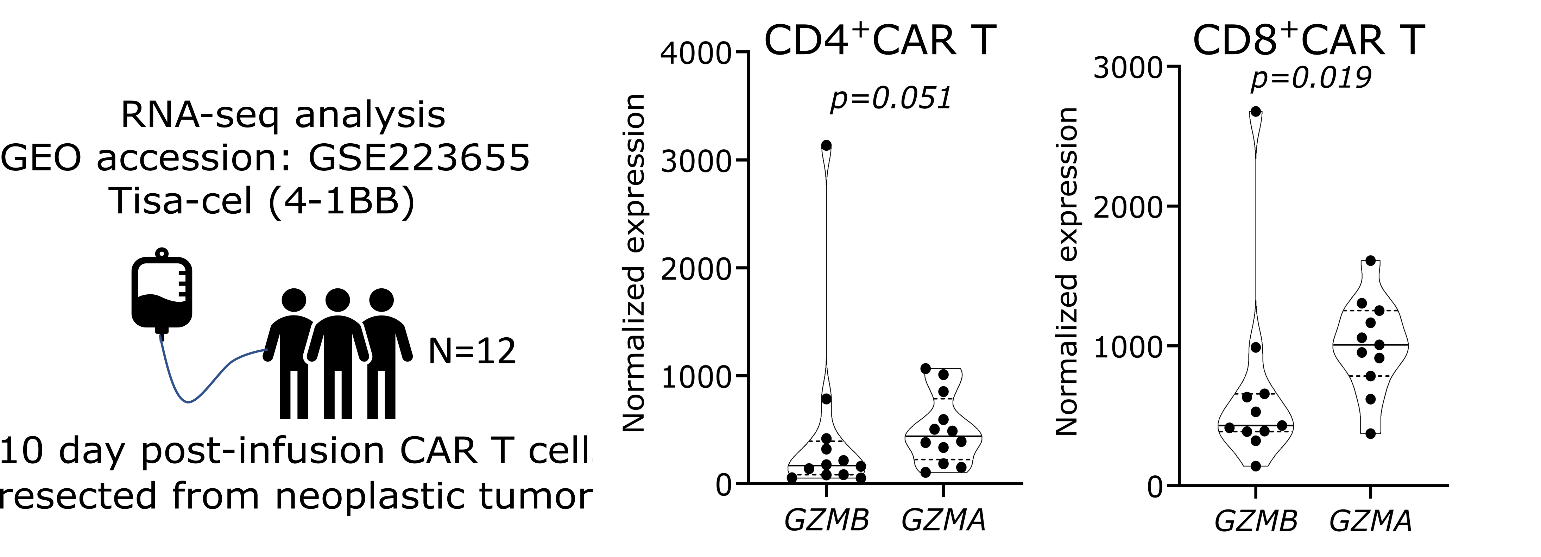


**Figure S15. Post-infusion Tisa-cel products show a higher expression of *GZMA* compared to *GZMB***. CAR T cells were isolated from DLBCL patient bone marrow after 10 days post-infusion and were sequenced based on CD4/CD8 classification. Statistical testing was performed using the Mann-Whitney test.
