# Supplementary figures and images for: Decoding the mechanisms of chimeric antigen receptor (CAR) T cell-mediated killing of tumors: insights from granzyme and Fas inhibition
Source: Cell Death Dis. 2024 Feb 2;15(2):109. doi: 10.1038/s41419-024-06461-8 (PMC10837176; doi:10.1038/s41419-024-06461-8)

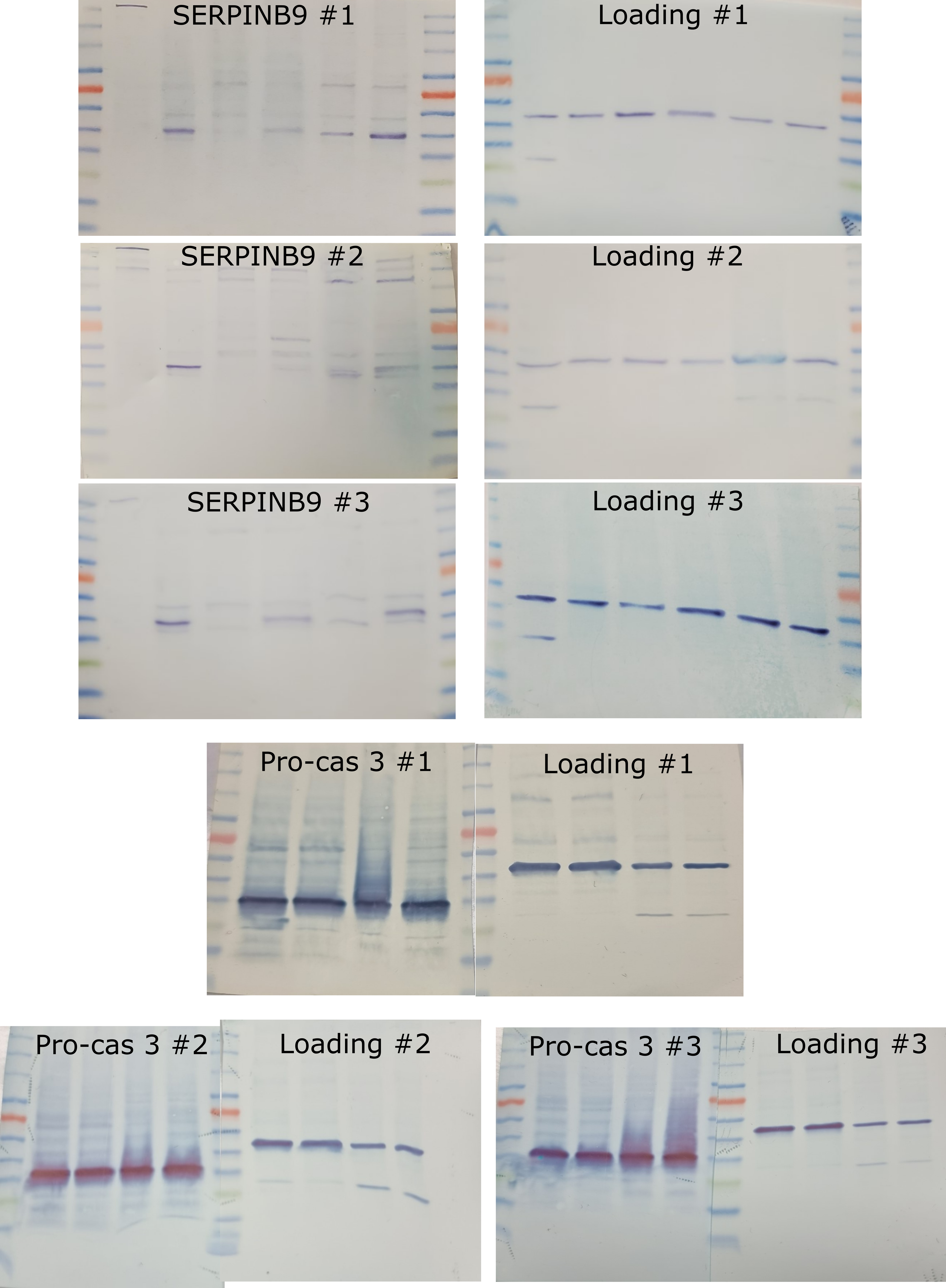

Supplement: Supplementary file 8 — Original Western blots [file 41419_2024_6461_MOESM8_ESM.png]
